# Intelectin 3 is dispensable for resistance against a mycobacterial infection in zebrafish (*Danio rerio*)

**DOI:** 10.1038/s41598-018-37678-1

**Published:** 2019-01-30

**Authors:** Markus J. T. Ojanen, Meri I. E. Uusi-Mäkelä, Sanna-Kaisa E. Harjula, Anni K. Saralahti, Kaisa E. Oksanen, Niklas Kähkönen, Juha A. E. Määttä, Vesa P. Hytönen, Marko Pesu, Mika Rämet

**Affiliations:** 1 Laboratory of Experimental Immunology, BioMediTech Institute and Faculty of Medicine and Life Sciences, University of Tampere, Tampere, Finland; 2 Laboratory of Immunoregulation, BioMediTech Institute and Faculty of Medicine and Life Sciences, University of Tampere, Tampere, Finland; 3 Laboratory of Protein Dynamics, BioMediTech Institute and Faculty of Medicine and Life Sciences, University of Tampere, Tampere, Finland; 4 Department of Dermatology, Tampere University Hospital, Tampere, Finland; 5 Department of Pediatrics, Tampere University Hospital, Tampere, Finland; 6 Department of Children and Adolescents, Oulu University Hospital, Oulu, Finland; 7 PEDEGO Research Unit and Medical Research Center Oulu, University of Oulu, Oulu, Finland

## Abstract

Tuberculosis is a multifactorial bacterial disease, which can be modeled in the zebrafish (*Danio rerio*). Abdominal cavity infection with *Mycobacterium marinum*, a close relative of *Mycobacterium tuberculosis*, leads to a granulomatous disease in adult zebrafish, which replicates the different phases of human tuberculosis, including primary infection, latency and spontaneous reactivation. Here, we have carried out a transcriptional analysis of zebrafish challenged with low-dose of *M. marinum*, and identified *intelectin 3* (*itln3*) among the highly up-regulated genes. In order to clarify the *in vivo* significance of Itln3 in immunity, we created nonsense *itln3* mutant zebrafish by CRISPR/Cas9 mutagenesis and analyzed the outcome of *M. marinum* infection in both zebrafish embryos and adult fish. The lack of functional *itln3* did not affect survival or the mycobacterial burden in the zebrafish. Furthermore, embryonic survival was not affected when another mycobacterial challenge responsive *intelectin*, *itln1*, was silenced using morpholinos either in the WT or *itln3* mutant fish. In addition, *M. marinum* infection in dexamethasone-treated adult zebrafish, which have lowered lymphocyte counts, resulted in similar bacterial burden in both WT fish and homozygous *itln3* mutants. Collectively, although *itln3* expression is induced upon *M. marinum* infection in zebrafish, it is dispensable for protective mycobacterial immune response.

## Introduction

Tuberculosis is an epidemic multifactorial disease caused by *Mycobacterium tuberculosis*^1^. The susceptibility to tuberculosis depends on the *M. tuberculosis* strain and on a number of host-related factors such as environmental conditions, other underlying diseases as well as genetic variation^2–4^. Critical genes of the adaptive immunity required for the mycobacterial immune response such as *interferon gamma* (*IFNG*)^5,6^ and *interleukin 12* (*IL12*)^7,8^ were identified already in the 1980’s and 1990’s, respectively. The importance of these genes has later been verified in human tuberculosis patients^9^ and by using experimental gene knockout mouse models of tuberculosis^10–13^. More recently, pattern recognition receptor (PRR) gene polymorphisms of Toll-like receptors (TLRs)^14–16^ and C-type lectins^17,18^, have been associated with *M. tuberculosis* susceptibility, delineating also the central role of the innate immunity in controlling the mycobacterial infection.

Lectins are carbohydrate-binding proteins important for numerous biological processes such as intracellular glycoprotein secretion, leukocyte trafficking and microbial recognition^19,20^. Consequently, lectins act as recognition molecules inside cells, on the cell surface and in extracellular fluids^20^. Intelectins (ITLNs) are a distinct family of lectins, which were first identified in *Xenopus laevis*^21^ and were later found in a number of chordates including human, mouse and zebrafish (*Danio rerio*)^22–25^. Although ITLN function has been linked to a number of processes such as iron absorption^26^, metabolic disorders^27^ as well as cancer development^28,29^, their exact biological functions are elusive. Suggesting a role for ITLNs in the immune response, *itln* gene expression is highly up-regulated upon a bacterial infection in fish^25,30–32^. Moreover, human ITLN1 (also known as Omentin) has been shown to bind to the *Mycobacterium bovis* Bacillus Calmette-Guérin (BCG)^33^, and more specifically to exocyclic 1,2-diol glycan epitopes that are expressed selectively on microbial surfaces^34^.

The importance of ITLNs for immunity *in vivo*, however, is less clear. Previously, Voehringer *et al*., used transgenic mice with lung-specific ITLN1 and ITLN2 over-expression to study the effects of these proteins in the mouse infection models of the parasite *Nippostrongylus brasiliensis* and the *M. tuberculosis* bacterium^35^. In these settings, the authors could not detect enhanced pathogen clearance in the *Itln* transgenic mice. In contrast, a so called “natural deletion” of the *Itln2* gene has been previously associated with a higher susceptibility against the parasite *Trichinella spiralis* in a C57BL/10 mouse strain^36^. Recently, an *Itln1* knockout mouse strain was created to study inflammatory bone diseases^37^. In the aforementioned study, the lack of Itln1 was associated with a proinflammatory phenotype characterized by elevated TNF and IL6 levels in bone tissue and in serum, and was shown to result in osteoporosis^37^.

The genome of the zebrafish was assembled for the first time in 2002 and the prevalent 11^th^ assembly (GRCz11) is an invaluable tool for research using zebrafish as a disease model^38^. Over 70% of human genes have at least one zebrafish orthologue and for this reason, the zebrafish immune system is highly similar compared to humans^38^. In fact, most of the human immune cell populations such as T- and B-cells^39–41^, neutrophils and macrophages^42^, dendritic cells^43^ as well as the complement system^44^ and immunoglobulins^45,46^, are found in the zebrafish. Importantly, zebrafish can be modified genetically with the clustered regularly interspaced short palindromic repeats (CRISPR)/CRISPR-associated 9 (Cas9) mutagenesis^47,48^, which allows disease modeling using reverse genetics, although some genes appear difficult to target successfully^49^.

A *Mycobacterium marinum* infection of zebrafish is nowadays a commonly used model for studying tuberculosis in both larvae and adult fish^50,51^. Compared to several other tuberculosis models, the mycobacterial model of zebrafish is considered safe, cost-effective and ethical^52,53^. More importantly, *M. marinum* is closely related to *M. tuberculosis*, and the two bacterial species have comparable pathogenic characteristics in the natural hosts; macrophage mediated intracellular multiplication as well as the formation of granuloma structures^54–56^. The larval model enables studying specifically the innate immunity^57,58^, whereas the adult zebrafish model allows studying also components of the adaptive immune system in both an acute mycobacterial infection^59^ as well as during mycobacterial latency^56,60^.

In order to identify candidate genes associated with the host response against mycobacteria, we conducted a gene expression microarray in *M. marinum* infected adult zebrafish. Here, we identified a zebrafish *ITLN* orthologue *itln3* among the genes that were most induced upon infection. In order to gain more insights into the function of ITLNs, we used CRISPR/Cas9 mutagenesis to create knockout *itln3* mutant zebrafish lines, and used the zebrafish *M. marinum* infection model to determine the *in vivo* significance of Itln3 in a mycobacterial infection.

## Results

### Genome-wide gene expression microarray analysis of *M. marinum* infected adult zebrafish

**Figure 1:**
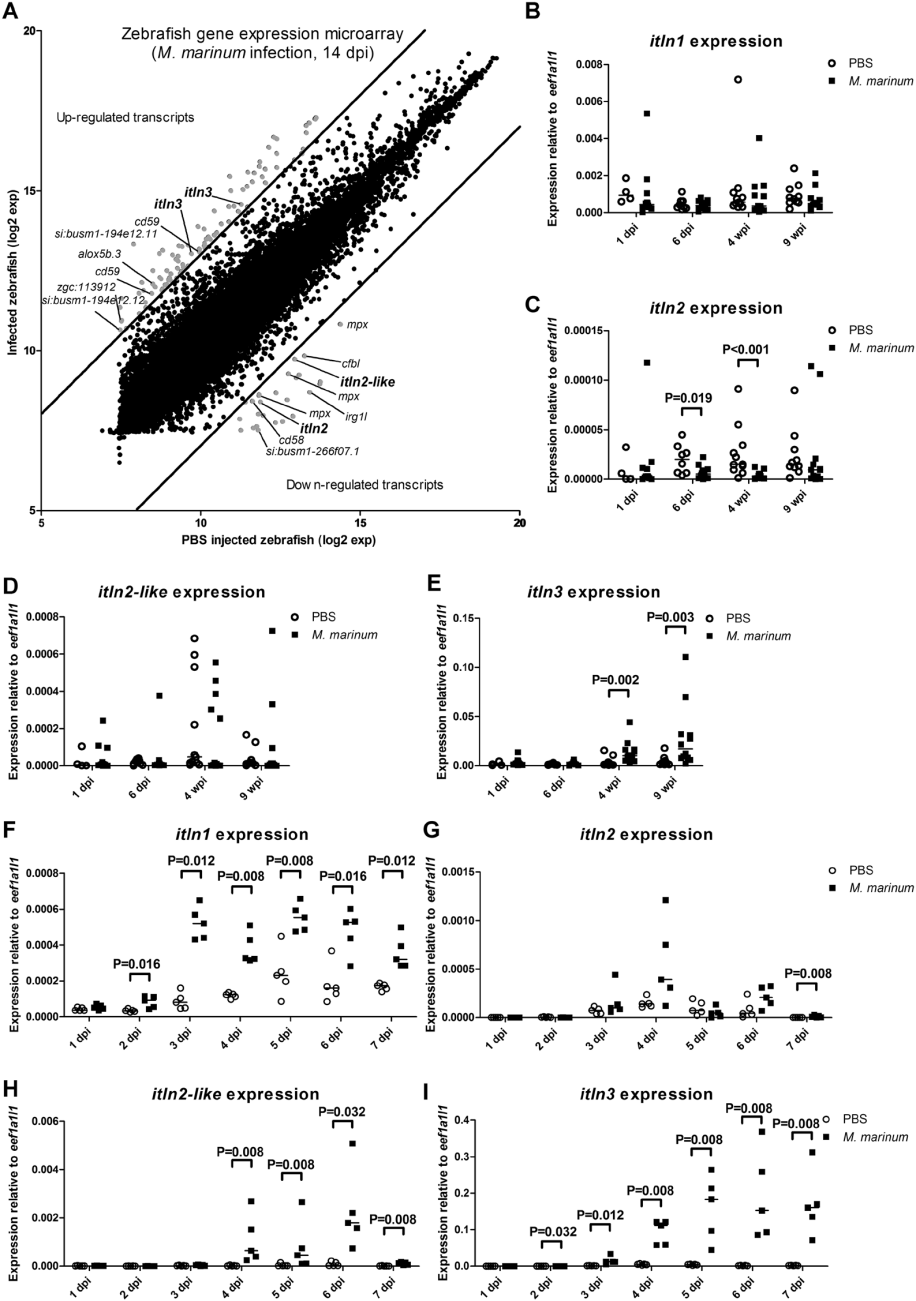
Zebrafish *intelectin* genes are differentially expressed upon *M. marinum* infection. (**A**) A genome-wide gene expression microarray was conducted in adult WT AB zebrafish injected with *M. marinum* (20 CFU; SD 6 CFU) (n = 2) or PBS (n = 3). Average numerical results (log2) for each probe in both infected fish (y-axis) and PBS controls (x-axis) are shown. Up- and down-regulated transcripts (log2 fold change $|3|$) in the organ blocks are shown in grey, and the common immunological genes are annotated. Two *itln3* probes as well as *itln2* and *itln2-like* probes are highlighted. (**B**–**E**) The expression of zebrafish *itln* genes (*itln1*, *itln2*, *itln2-like* and *itln3*) was measured with qPCR in the organ blocks of the *M. marinum* infected (6 CFU; SD 3 CFU) and PBS injected adult WT e46 zebrafish at 1 (n = 12 and n = 4, respectively) and 6 dpi (n = 12 and n = 8, respectively) as well as 4 (n = 12 and n = 11, respectively) and 9 wpi (n = 12 and n = 10, respectively). (**F**–**I**) The expression of *itln1*, *itln2*, *itln2-like* and *itln3* was determined with qPCR in the *M. marinum* (39 CFU; SD 47 CFU) infected WT AB embryos (n = 5 at all timepoints) and in PBS injected controls (n = 5 at all timepoints) at 1–7 dpi. Note the different scales of the y axes in B-I. Gene expressions were normalized to *eef1a1l1* expression and target genes were run once in the qPCR analyses. A two-tailed Mann-Whitney test was used in the statistical comparison of differences in B–I.

In order to identify genes involved in the host immune response against mycobacteria, we used the zebrafish *M. marinum* infection model and conducted a genome-wide gene expression analysis using the microarray platform. To this end, we infected wild-type (WT) AB zebrafish with *M. marinum* (20 CFU; SD 6 CFU) and isolated their organ blocks (includes all the organs of the abdominal cavity) for a transcriptomic analysis at 14 days post infection (dpi). From a total of 43603 probes used in the analysis, we found 93 probes, corresponding to 70 genes, that were up-regulated and 26 probes, corresponding to 21 genes, that were down-regulated (log2 fold change >$|3|$) compared to the mock-treated (PBS) controls (Supplementary Table 1). Further evaluation of the up-regulated probes with a GOrilla gene ontology (GO) enrichment analysis^61,62^ revealed 22 enriched (p < 0.001) processes including response to carbohydrates (GO:0009743), cholesterol homeostasis (GO:0042632) and antigen processing and presentation (GO:0019882) (Supplementary Table 2). Among the up-regulated genes we found five genes; *si:busm1-194e12.11* (*mhc2* family gene), *arachidonate 5-lipoxygenase b, tandem duplicate 3* (*alox5b.3*), *zgc:113912* (*mhc2* family gene), *CD59 molecule* (*cd59*) and *si:busm1-194e12.12* (*mhc2* family gene) with well-known immunological functions in antigen processing, inflammation and in the regulation of the complement system (Fig. 1A, Supplementary Table 1). Of the 21 down-regulated genes, five were associated with the immune response; *CD58 molecule* (*cd58*), *myeloid-specific peroxidase* (*mpx*), *complement factor b-like* (*cfbl*), *immunoresponsive gene 1, like* (*irg1l*) and *si:busm1-266f07.1* (*mhc2* family gene) (Fig. 1A, Supplementary Table 1). Interestingly, approximately 38% of the up-regulated probes i.e. *parvalbumin 1* (*pvalb1*), *alpha-tropomyosin* (*tpma*), *troponin I, skeletal, fast 2b, tandem duplicate 2* (*tnni2b.2*) and *myosin, heavy polypeptide 1.1* (*myhz1.1*) were related to muscle associated biological processes including muscle contraction (GO:0006936), muscle system process (GO:0003012) and myofibril assembly (GO:0030239) (Supplementary Tables 1 and 2). The GO-analysis of the down-regulated probes also showed a significant enrichment of another 22 processes including response to external biotic stimulus (GO: 0043207) and cholesterol biosynthetic process (GO:0006695) and immunological processes, such as response to other organism (GO:0051707), response to bacterium (GO:0009617) and the induction of bacterial agglutination (GO:0043152) (Supplementary Table 2).

### Mycobacterial infection up-regulates *itln3* expression in both zebrafish embryos and adult fish

Previous studies in several animal models have shown the expression of the *Intelectin* (*ITLN*) gene to be induced upon a bacterial infection^25,30,32^. Accordingly, the expression of the zebrafish *itln3* (ENSDARG00000003523) was increased on average 3.3-fold (log2 change) upon a *M. marinum* infection in our microarray analysis (Fig. 1A, Supplementary Table 1). In contrast, two other *itln* genes; *itln2* (ENSDARG00000036084) and *itln2-like* (ENSDARG00000093796) were down-regulated compared to the PBS controls (−3.5 and −3.2 log2 fold change, respectively) (Fig. 1A, Supplementary Table 1), suggesting a diverse regulation of *itln* genes in the *M. marinum* infected zebrafish. Since both ENSDARG00000036084 and ENSDARG00000093796 share the same gene name, *itln2*, in Ensembl genome browser, ENSDARG00000093796 is referred to as *itln2-like* throughout the text.

To confirm the differential expression pattern of the *itln* family members in the zebrafish mycobacterial infection and to study the kinetics of the host response more carefully, we analyzed *itln1* (ENSDARG00000007534), *itln2*, *itln2-like* and *itln3* gene expression from the abdominal cavity organ blocks of *M. marinum* infected (6 CFU; SD 3 CFU) WT e46 background adult zebrafish with qPCR at 1 and 6 dpi, as well as at 4 and 9 weeks post infection (wpi) (Fig. 1B–E). In line with our microarray data, *itln3* was significantly induced at 4 wpi (3.8-fold, P = 0.002) and 9 wpi (5.9-fold, P = 0.003) (Fig. 1E), whereas *itln2* was down-regulated compared to the PBS controls both at 6 dpi (0.3-fold, P = 0.019) and 4 wpi (0.2-fold, P < 0.001) (Fig. 1C). No significant differences in the relative mRNA expression levels of the *itln1* (Fig. 1B) or *itln2-like* (Fig. 1D) genes were observed between infected and the PBS injected adult fish at any of the measured time points.

Next, we infected WT AB zebrafish embryos with mycobacteria and performed an expression analysis of the *itln* genes by qPCR. Here, *M. marinum* (39 CFU; SD 47 CFU) was microinjected into the yolk sac of the embryos and the gene expression was quantified daily between 1 and 7 dpi (Fig. 1F–I). In the mycobacteria infected embryos we detected the up-regulation of both *itln1* (1.8 to 6.4-fold, P = 0.008–0.016) (Fig. 1F) and *itln3* (1.8 to 111.4-fold, P = 0.008–0.032) (Fig. 1I) starting at 2 dpi and continuing until 7 dpi, as well as the induction of *itln2* at 7 dpi (21.6-fold, P = 0.008) (Fig. 1G) and *itln2-like* (Fig. 1H) between 4 and 7 dpi (20.7 to 76.1-fold, P = 0.008–0.032) compared to the PBS controls. Also, in line with previous reports suggesting that other infectious diseases up-regulate *ITLN* expression, a significant induction of *itln3* expression (8.4-fold at 7hpi; 11.8-fold at 18hpi; 5.5-fold at 24hpi; 4.4-fold at 48hpi, P = 0.002 in all comparisons) was observed in *Streptococcus pneumoniae* (T4 serotype) infected (296 CFU; SD 32 CFU) embryos (Supplementary Figure 1).

**Figure 2:**
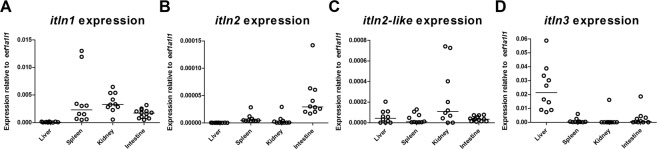
Expression of zebrafish *itln* genes in adult zebrafish tissues. Relative expression of (**A**) *itln1*, (**B**) *itln2*, (**C**) *itln2-like* and (**D**) *itln3* was measured with qPCR in the uninfected adult WT e46 zebrafish liver (n = 10), spleen (n = 10), kidney (n = 10) and the intestine (n = 10). Note the different scales of the y axes. Gene expressions were normalized to *eef1a1l1* expression and target genes were run once in the qPCR analyses.

In order to understand the infection-inducible nature of the zebrafish *itln* genes at steady state, we quantified the relative mRNA levels of *itln1*, *itln2*, *itln2-like* and *itln3* in the liver, spleen, kidney and intestine of unchallenged WT e46 zebrafish by qPCR (Fig. 2A–D). Here, we found that *itln2* expression was restricted to the intestine (Fig. 2B), whereas *itln3* showed the highest relative expression in the liver and the highest overall expression compared to the housekeeping gene (*eukaryotic translation elongation factor 1 alpha 1, like 1; eef1a1l1*) (Fig. 2D). Conversely, *itln1* was expressed in all of the studied tissues with the second highest overall expression levels (Fig. 2A), while *itln2-like* was primarily expressed in the zebrafish kidney and the intestine (Fig. 2C). These results are in line with a previous qPCR analysis of the *itln* gene family members in unchallenged adult zebrafish^25^.

### Creating *itln3* mutant zebrafish using CRISPR/Cas9 mutagenesis

**Figure 3:**
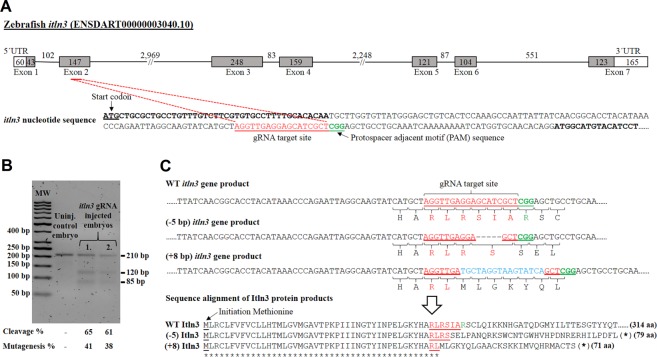
Generation of *itln3*^uta145^ and *itln3*^uta148^ mutant zebrafish lines using CRISPR-Cas9 mutagenesis. (**A**) An appropriate guide RNA (gRNA) target site was identified in the second exon of *itln3*. (**B**) 2.5% agarose TAE gel electrophoresis was performed to evaluate occurrence of target site mutations in zebrafish. The *in vivo* CRISPR/Cas9 mutagenesis efficiency was estimated with the T7EI assay in the gRNA and *Cas9* mRNA injected embryos. The size of the uninjected WT control PCR product is 210 bp, whereas the PCR products of the mutated embryos are partially cleaved at the target site. The cleavage efficiency was calculated from the band intensities and the mutagenesis efficiency calculated according to the following formula: % mutagenesis = 100 × (1 – (1- fraction of cleavage)^1/2^)^64^. GeneRuler 50 bp DNA Ladder (#SM0373, Thermo Fischer Scientific) was used as a molecular weight marker (MW). Gel image is cropped to exclude portions that do not contain experimental samples. (**C**) gRNA target sites were sequenced from F1-generation mutant zebrafish and two frameshift mutations (-5 bp deletion, *itln3*^uta145^ and +8 bp insertion, *itln3*^uta148^) detected, leading to truncated protein products of 79 and 71 amino acids, respectively.

The type II CRISPR/Cas system is an invaluable technology for targeted genome editing^63,64^, and to date it has been utilized in a number of model organisms. We and others have used the CRISPR/Cas9 mutagenesis method successfully in the zebrafish^47,49,65,66^. Here, we used the CRISPR/Cas9 method to create zebrafish carrying nonsense *itln3* mutations for our *in vivo* studies (Fig. 3). To this end, we identified a functional gRNA targeting the second exon of the *itln3* gene with an average mutagenesis efficiency of 39.5% (Fig. 3A,B). After an outcross of parental mutation carriers (F0-generation) with WT TL zebrafish, we observed two germ-line transmitted frameshift mutations in the F1-progeny corresponding to a total loss of five base pairs (−5 bp; loss of GCATC) and to a total gain of eight base pairs (+8 bp; loss of GGAGCATC and gain of TGCTAGGTAAGTATCA) at the target loci (Fig. 3C). Analyses with the Translate tool (Expasy; SIB, Swiss Institute of Bioinformatics)^67^ of both the −5 bp and +8 bp mutations confirmed the disrupted reading frames from amino acids 47 and 45 onwards resulting in premature stop-codons after 79 and 71 amino acids, respectively (Fig. 3C). These two different *itln3* null mutant zebrafish lines were named *itln3*^uta145^ (−5 bp mutation) and *itln3*^uta148^ (+8 bp mutation). qPCR analysis of uninjected and *M. marinum* infected (422 CFU; SD 221 CFU, 2 wpi) adult zebrafish revealed diminished *itln3* transcript levels in the homozygous *itln3*^uta145/uta145^ (residual expression less than 1%, P < 0.001) and *itln3*^uta148/uta148^ mutants (residual expression less than 0.1%, P < 0.001) compared to the WT controls (Supplementary Figure 2), suggesting that the indel-mutations lead to the nonsense-mediated RNA decay of the mutant mRNAs^68^. Furthermore, the inheritance of the mutations followed Mendelian ratios for both of the mutant lines, and the homozygous *itln3*^uta145/uta145^ and *itln3*^uta148/uta148^ mutants did not show any developmental defects nor phenotypical differences compared to their WT siblings (Supplementary Figure 3).

### Nonsense mutation in *itln3* does not affect host resistance against *M. marinum* in zebrafish embryos

**Figure 4:**
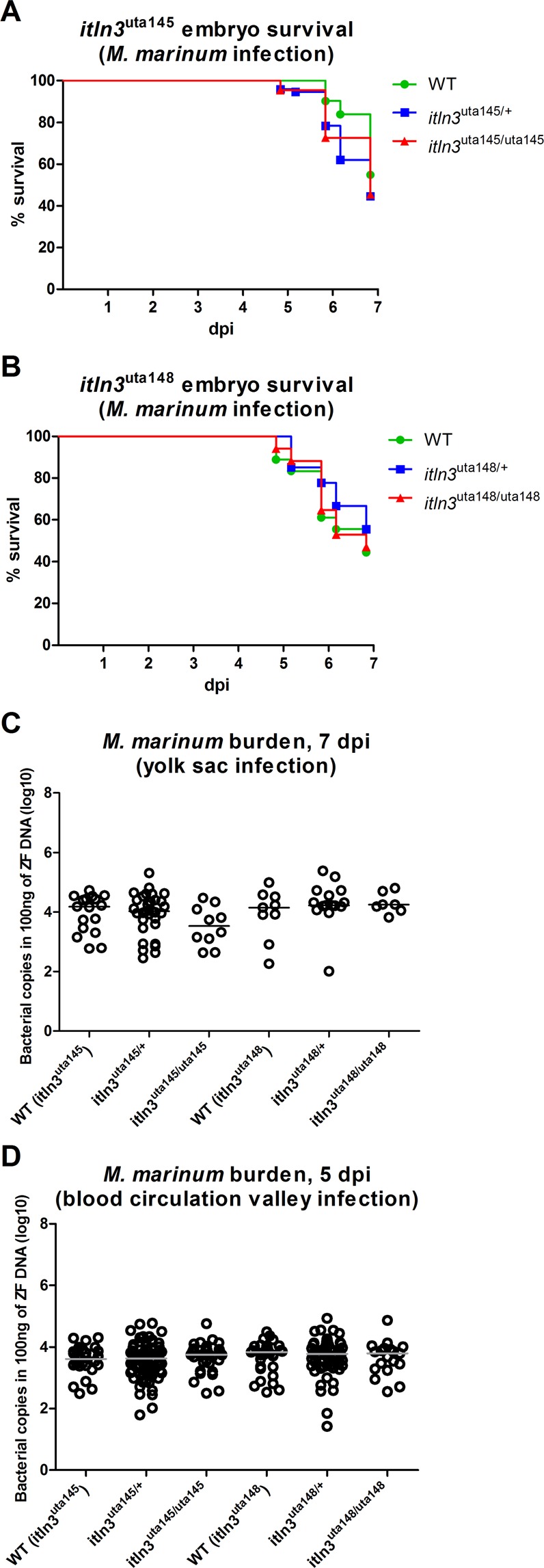
The lack of *itln3* does not affect the survival or the mycobacterial burden of *M. marinum* infected zebrafish embryos. (**A,B**) *M. marinum* (40 CFU; SD 30 CFU) was injected into the yolk sac of the WT (*itln3*^uta145^) (n = 31), *itln3*^uta145/+^ (n = 74), *itln3*^uta145/uta145^ (n = 22), WT (*itln3*^uta148^) (n = 19), *itln3*^uta148/+^ (n = 27) and *itln3*^uta148/uta148^ (n = 16) zebrafish embryos at 0 dpf and the survival recorded until 7 dpi. A log-rank (Mantel-Cox) test was used for the statistical comparison of differences. The data was collected from a single experiment. (**C**) Mycobacterial burden was measured by qPCR from the yolk sac infected WT (*itln3*^uta145^) (n = 17), *itln3*^uta145/+^ (n = 32), *itln3*^uta145/uta145^ (n = 10), WT (*itln3*^uta148^) (n = 9), *itln3*^uta148/+^ (n = 15) and *itln3*^uta148/uta148^ (n = 7) embryos that were alive at 7 dpi. (**D**) *M. marinum* (46 CFU; SD 31 CFU) was injected into the blood circulation valley of the WT (*itln3*^uta145^) (n = 29), *itln3*^uta145/+^ (n = 77), *itln3*^uta145/uta145^ (n = 36), WT (*itln3*^uta148^) (n = 31), *itln3*^uta148/+^ (n = 57) and *itln3*^uta148/uta148^ (n = 19) zebrafish embryos at 2 dpf and the *M. marinum* burden quantified at 5 dpi. Bacterial load is represented in panels C and D as bacterial copies (log10) in 100 ng of zebrafish DNA. A two-tailed Mann-Whitney test was used in the statistical comparison of differences in C and D.

The up-regulation of the expression of the *itln3* gene in a *M. marinum* infection suggests a possible role for Itln3 in the host immunity against mycobacterial infections. To test if the resistance towards a mycobacterial infection is altered in homozygous *itln3* mutant embryos, we first infected *M. marinum* (40 CFU; SD 30 CFU) into the yolk sac of the ungenotyped F2-progeny of heterozygous *itln3*^uta145/+^ and *itln3*^uta148/+^ zebrafish and followed their survival until 7 days post fertilization (dpf) (Fig. 4A,B). Post-experiment genotyping revealed an average survival of 47% in the *itln3*^uta145^ background embryos and 48% in the embryos with the *itln3*^uta148^ background. However, any significant differences in the survival between the homozygous and heterozygous *itln3* mutants or WT fish could not be observed in either *itln3*^uta145^ (Fig. 4A) or *itln3*^uta148^ fish lines (Fig. 4B) before 7 dpi (7 dpf). Next, we quantified the mycobacterial burden in the embryos that had survived by qPCR using primers for *M. marinum internal transcribed spacer* (*MMITS*)^56^ (Fig. 4C). The *M. marinum* quantification revealed bacterial copy number medians (log10) of 4.18 and 4.15 in 100 ng of zebrafish DNA in the *itln3*^uta145^ and *itln3*^uta145^ WT groups, respectively. Comparably, heterozygous *itln3*^uta145/+^ and *itln3*^uta148/+^ fish had copy number medians of 4.02 and 4.22 (in 100 ng of zebrafish DNA, log10), respectively, and the homozygous *itln3*^uta145/uta145^ and *itln3*^uta148/uta148^ mutants 3.53 and 4.25 (in 100 ng of zebrafish DNA, log10). Thus, there were no statistically significant differences in the mycobacterial burdens between the different genotypes in neither *itln3*^uta145^ nor *itln3*^uta148^ zebrafish.

The site of the bacterial injection can affect the immune response in the embryos^50^. Therefore, we next treated the ungenotyped F2-progeny of *itln3*^uta145/+^ and *itln3*^uta148/+^ zebrafish by injecting *M. marinum* into the blood circulation valley of 2-day-old embryos. In these fish, the *M. marinum* infection (46 CFU; SD 31 CFU) was not able to cause any mortality prior to the experimental end-point of 5 dpi (7 dpf). However, this allowed us to quantify the *M. marinum* burden in all of the infected embryos at the end-point (Fig. 4D). Here, the bacterial copy number medians (log10) in 100 ng of zebrafish DNA were 3.61 (WT *itln3*^uta145^), 3.83 (WT *itln3*^uta148^), 3.63 (*itln3*^uta145/+^), 3.77 (*itln3*^uta148/+^), 3.75 (*itln3*^uta145/uta145^) and 3.79 (*itln3*^uta148/uta148^). Similarly to the yolk sac infection, mycobacterial quantification did not reveal any differences between the individuals of the different genotypes in either the *itln3*^uta145^ or the *itln3*^uta148^ zebrafish background. Noteworthy, we also infected the ungenotyped F2-progeny of *itln3*^uta145/+^ and *itln3*^uta148/+^ zebrafish with S. *pneumoniae* (serotypes 1 and T4, blood circulation valley infection at 2 dpf) and followed the survival of the fish to 5 dpi^69^. There was no difference between WT embryos and the *itln3* mutants (Supplementary Figure 1).

**Figure 5:**
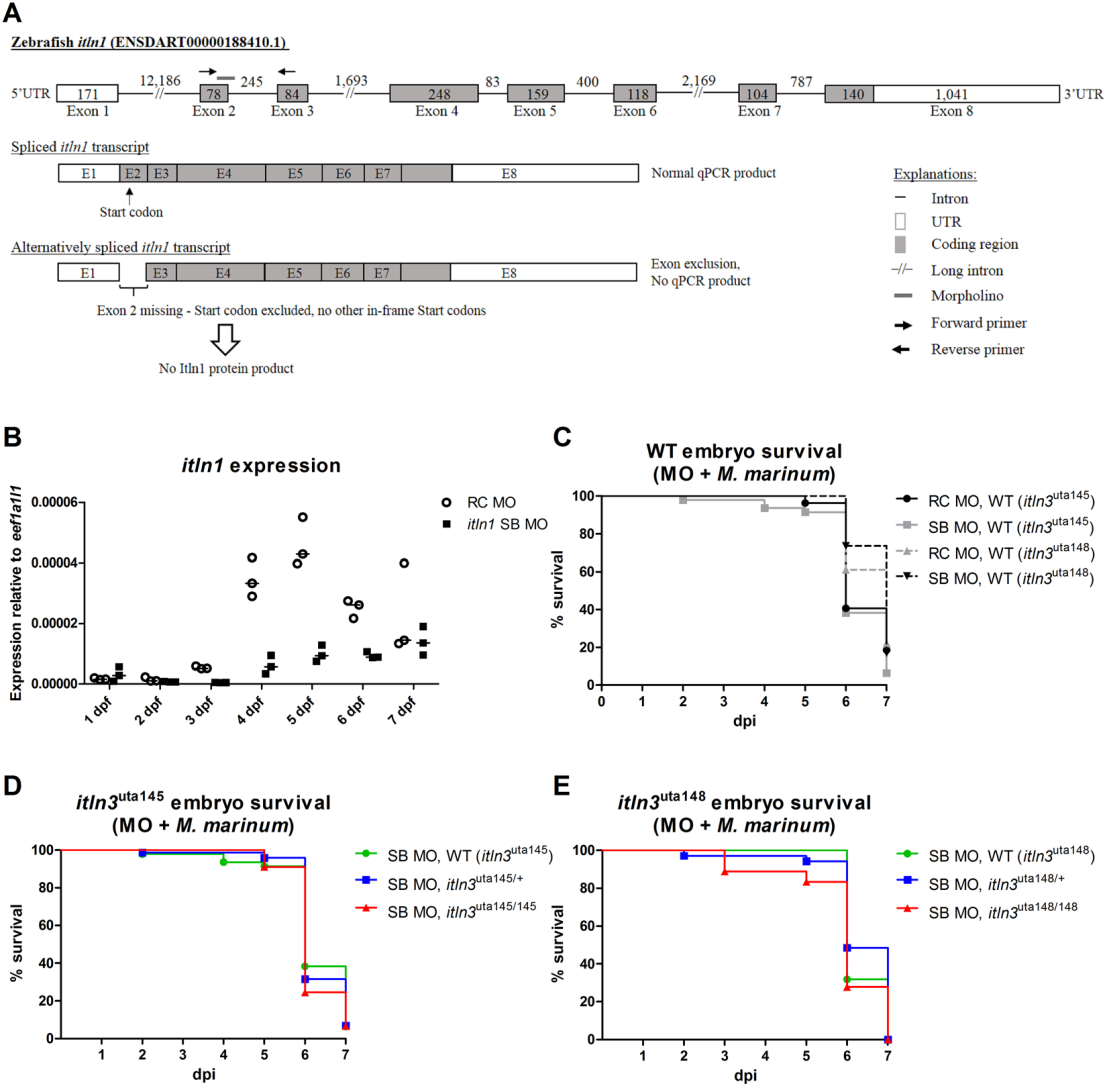
Morpholino mediated silencing of *itln1* expression does not alter the survival of the WT or *itln3* knockout zebrafish in a *M. marinum* infection. (**A**) A schematic representation of the effects of the morpholino mediated silencing of *itln1*. A splice site blocking morpholino (SB) was used to prevent the normal splicing event between exon 2 and exon 3 in *itln1*. Morpholino binding to its target site leads to an alternative splicing event that deletes the start codon containing exon 2 from the transcript. Consequently, this prevents translation of the Itln1 protein. In order to quantify the relative amount of the WT *itln1* transcript, qPCR primers were designed to specifically amplify only the WT *itln1* mRNA. (**B**) WT *itln1* expression was quantified with qPCR from the *itln1* SB morpholino (n = 3) and random control morpholino (RC) injected zebrafish (n = 3) at 1–7 dpf. Gene expression was normalized to *eef1a1l1* expression. All samples were run once as technical duplicates. (**C**–**E**) Survival of the morpholino and *M. marinum* (20 CFU; SD 19 or 13 CFU; SD 10 CFU) co-injected embryos were followed until 7 dpi. In panel C, WT (*itln3*^*uta145*^ and *itln3*^*uta148*^) embryos injected with either SB (n = 47 and n = 53, respectively) or RC morpholino (n = 29 and n = 54) are shown, whereas in panels D and E the *itln3*^*uta145*^ background (n = 45–73) and *itln3*^*uta148*^ background embryos (n = 18–35) injected with SB morpholino are depicted, respectively. Note that the SB morpholino injected WT (*itln3*^*uta145*^) embryo group is shown in both C and D panels in order to simplify data representation. The data in panel C was collected from two individual experiments, whereas other data is from a single experiment. A log-rank (Mantel-Cox) test was used for the statistical comparison of differences. MO = morpholino.

Deleterious mutations may lead to genetic compensation, which in turn can affect the observed phenotype in gene knockout models^70^. To address this, we used a morpholino-oligonucleotide to silence *itln1* in our *itln3* mutant zebrafish together with the yolk sac mycobacterial infection of zebrafish embryos. In order to ensure efficient termination of translation in all of the four zebrafish *itln1* transcripts, we targeted the second exon (E2) of the gene with a splice-blocking (SB) morpholino (Fig. 5A). In our initial SB morpholino titration experiments, 2.8 ng of *itln1*-blocking morpholino did not reveal any adverse effects on the survival or on the phenotype of unchallenged zebrafish embryos within the first 7 dpf. However, lower WT *itln1* mRNA levels were observed in the SB morphants with residual expression of 17.1% at 4 dpi, 21.8% at 5 dpi and 33.9% at 6 dpi compared to the random control (RC) injected embryos (Fig. 5B), demonstrating that this amount of the SB morpholino silences the expression of *itln1* efficiently during embryonic development. In addition, detectable *itln1* expression levels were observed in the RC morphants already at 1 dpi, whereas in the SB morpholino injected embryos *itln1* expression was evident later starting at 2 dpi based on qPCR (Fig. 5B, Supplementary Figure 4). Next, we performed morpholino-*M. marinum* co-injections (20 CFU; SD 19 CFU) into the yolk sac of the un-genotyped F2-progeny of *itln3*^uta145/+^ and *itln3*^*uta148/+*^ zebrafish (Fig. 5C–E) and the F3-progeny of WT *itln3*^uta148^ (13 CFU; SD 10 CFU) (Fig. 5C) and followed their survival up to 7 dpi. There were few dying embryos among uninfected embryos upon RC or *itln1* morpholino injection (Supplementary Figure 4), whereas the mortality reached 77.8–100% in the morpholino-*M. marinum* co-injected embryos. Noteworthy, the comparison between the infected RC and SB morpholino injected WT *itln3*^uta145^ and *itln3*^uta148^ embryos did not show any differences in survival (Fig. 5C). Moreover, inhibiting *itln1* expression in homozygous *itln3*^uta145/uta145^ and *itln3*^uta148/uta148^ mutants lead to a similar mortality compared to the corresponding heterozygous and WT siblings of the same genetic background (Fig. 5D,E), indicating that the simultaneous lack of *itln1* and *itln3* functionality does not affect mycobacterial resistance in the zebrafish embryo. Consistently, we did not detect any differences in the mRNA levels of *itln1*, *itln2* and *itln2-like* between the homozygous *itln3* mutants and the WT controls either in uninjected (4 dpf) or *M. marinum* (25 CFU; SD 23 CFU, 4 dpf/4 dpi) infected embryos (Supplementary Figure 5), suggesting that there is no transcriptional compensation by the other studied *intelectin* gene members in the *itln3*^uta145/uta145^ and *itln3*^uta148/uta148^ mutant fish. Similarly, no transcriptional compensation by *itln1*, *itln2* or *itln2-like* was observed in the adult *itln3* mutant zebrafish either in steady state or upon *M. marinum* infection (Supplementary Figure 2).

### Adult *itln3* mutant zebrafish have a normal immune response towards a *M. marinum* infection

**Figure 6:**
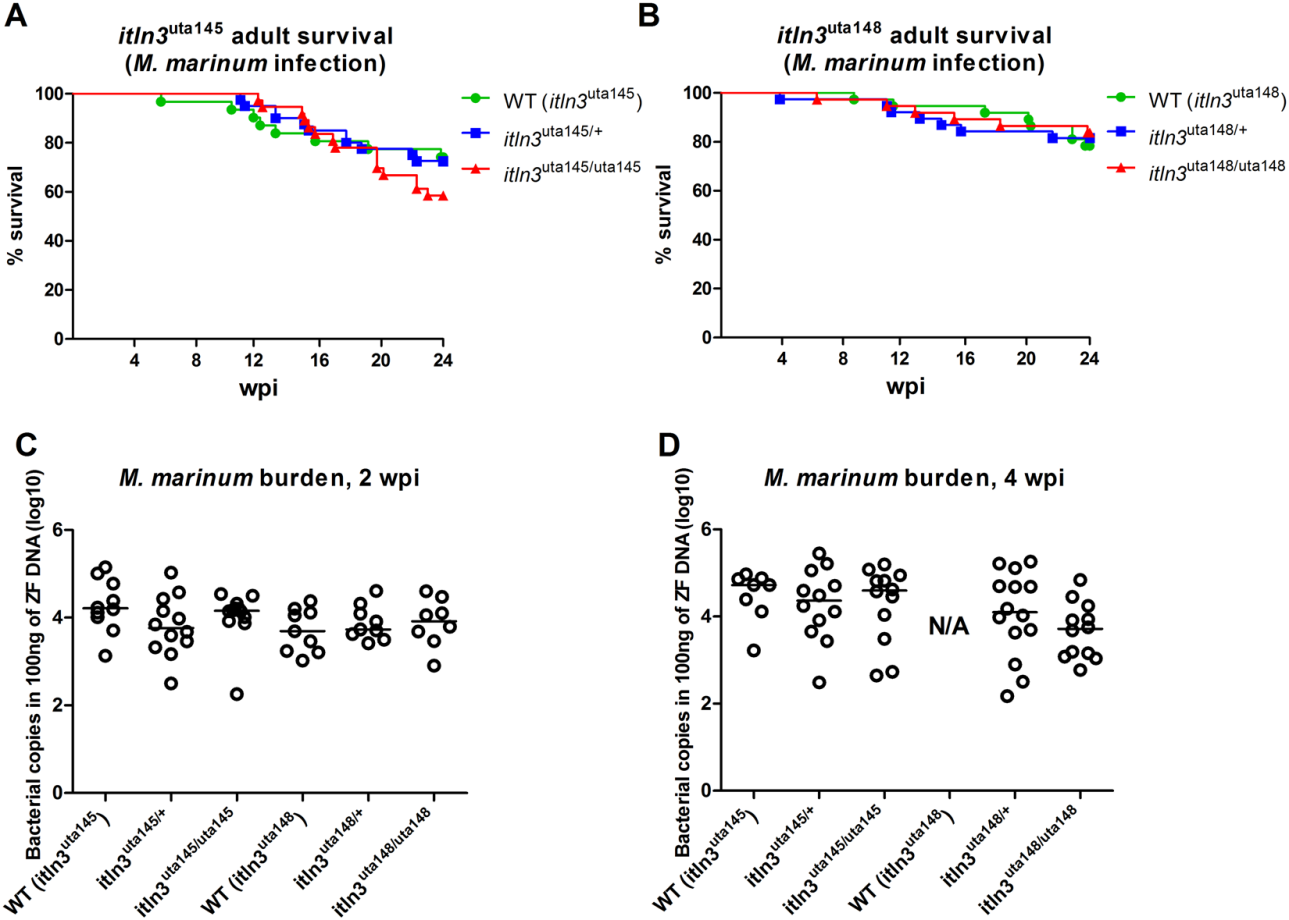
Adult *itln3* mutant zebrafish have comparable survival and mycobacterial burden compared to WT fish upon *M. marinum* infection. (**A**) The WT (*itln3*^*uta145*^) (n = 38), *iltn3*^uta145/+^ (n = 40), *iltn3*^uta145/uta145^ (n = 38) and (**B**) WT (*itln3*^*uta148*^) (n = 38), *iltn3*^uta148/+^ (n = 38), *iltn3*^uta148/uta148^ (n = 38) zebrafish were infected with *M. marinum* (48 CFU; SD 5), and their survival followed for 24 weeks. A log-rank (Mantel-Cox) test was used for the statistical comparison of differences. The data was collected from a single experiment. (**C**,**D**) The *itln3*^uta145^ and *itln3*^uta148^ background zebrafish were infected with *M. marinum* (422 CFU; SD 221 CFU) and bacterial burden (log10) in 100 ng of zebrafish DNA determined at 2 and 4 wpi from the organ blocks (without the kidney). Group sizes at 2 and 4 wpi, respectively, were as follows: WT (*itln3*^*uta145*^) n = 10, n = 8; *iltn3*^uta145/+^ n = 12, n = 12; *iltn3*^uta145/uta145^ n = 12, n = 12; WT (*itln3*^*uta148*^) n = 9, n = N/A; *iltn3*^uta148/+^ n = 9, n = 14 and *iltn3*^uta148/uta148^ n = 8, n = 12. All samples were run once. A two-tailed Mann-Whitney test was used in the statistical comparison of differences. N/A = no fish available for analysis.

In order to test the mycobacterial susceptibility of the *itln3* mutants in adult zebrafish, we performed a low-dose (48 CFU; SD 5 CFU) mycobacterial inoculation into the abdominal cavity of the fish and followed their survival for up to 24 wpi (Fig. 6A,B). After the follow-up, an average of 67% of the *itln3*^uta145^ background zebrafish had survived, corresponding to 74% of the WT, 73% of the *itln3*^uta145/+^ and 59% of the *itln3*^uta145/uta145^ fish. In the *itln3*^uta148^ background fish, a combined survival percentage of 81% was observed (78% in the WT, 82% in the *itln3*^uta148/+^ and 84% in the *itln3*^uta148/uta148^ fish). Similarly to the embryonic survival experiments, no statistically significant differences in the survival between the genotypes were observed.

We and others have previously shown that the outcome of a mycobacterial infection in adult zebrafish depends not only on the host genotype but also on the infection dose. While, a so called low-dose inoculate can result in latency and a chronic disease^56^, a higher dose leads to a fast progressing acute infection^59,71^. We hypothesized that the effects caused by the lack of Itln3 could be more prominent in an infection with a higher mycobacterial dose. Consequently, we infected WT fish as well as heterozygous and homozygous *itln3* mutants from both the *itln3*^uta145^ and *itln3*^uta148^ backgrounds with a higher *M. marinum* dose (422 CFU; SD 221 CFU) and quantified the bacterial burden at 2 and 4 wpi (Fig. 6C,D). In these fish, we detected *M. marinum* copy number medians (log10) of 4.19 (WT *itln3*^uta145^), 3.76 (*itln3*^uta145/+^), 4.16 (*itln3*^uta145/uta145^), 3.69 (WT *itln3*^uta148^), 3.73 (*itln3*^uta148/+^) and 3.92 (*itln3*^uta148/uta148^) in 100 ng of zebrafish DNA at 2 wpi and 4.72 (WT *itln3*^uta145^), 4.36 (*itln3*^uta145/+^), 4.60 (*itln3*^uta145/uta145^), 4.10 (*itln3*^uta148/+^) and 3.72 (*itln3*^uta148/uta148^) at 4 wpi. Noteworthy, no WT *itln3*^uta148^ fish were available at 4wpi for a bacterial quantification. Altogether, these data indicate that the loss of Itln3 function is dispensable for the host resistance against abdominal cavity *M. marinum* infection in adult zebrafish.

### Dexamethasone mediated lymphocyte depletion in *itln3* knockout zebrafish does not affect the survival or mycobacterial burden in a *M. marinum* infection

**Figure 7:**
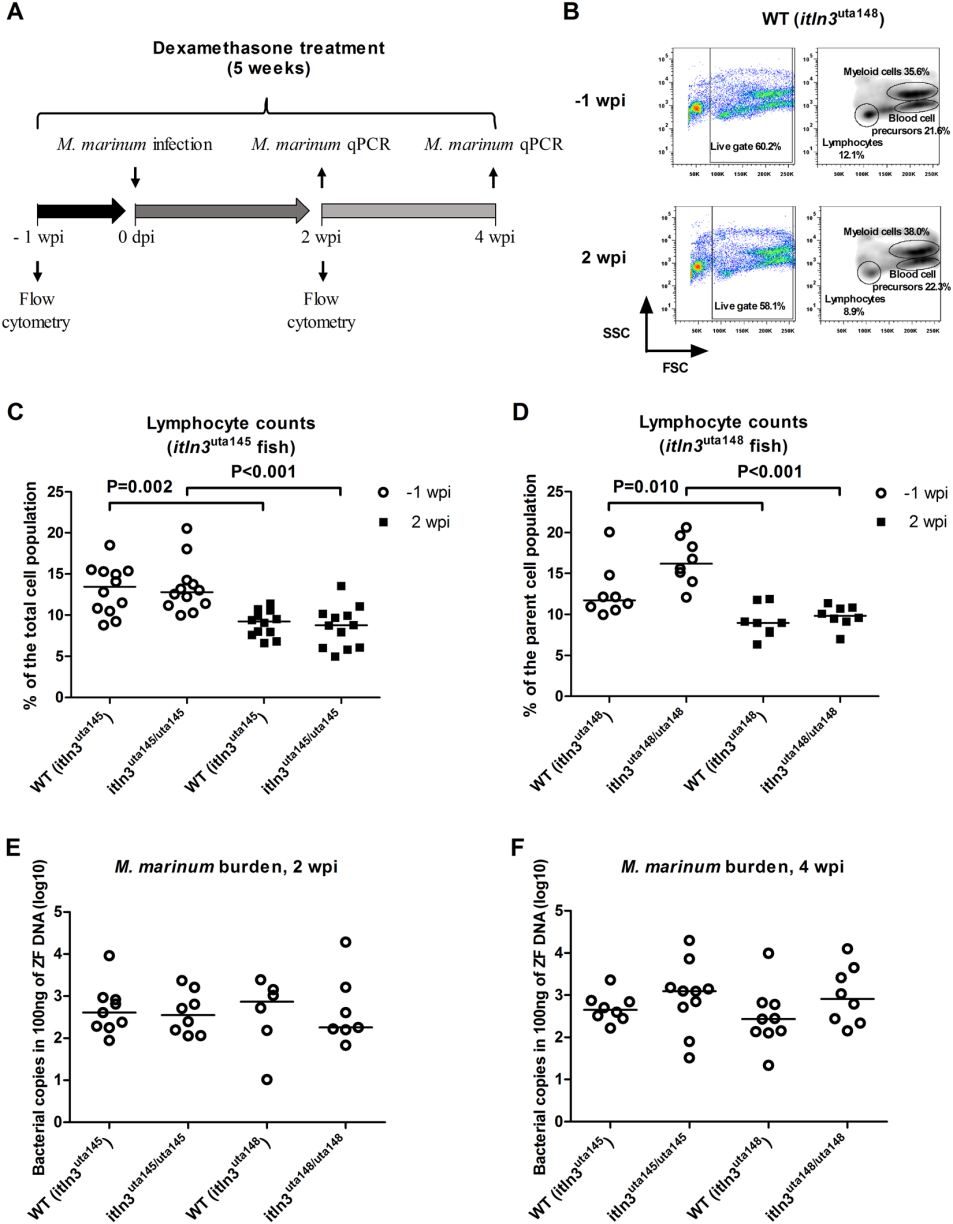
Dexamethasone mediated immunosuppression does not alter the survival of *itln3* deficient zebrafish in a mycobacterial infection. (**A**) A schematic representation of the performed experiment. *M. marinum* inoculate used in the infections were 47 CFU (SD 4 CFU). (**B**) Representative flow cytometry plots in WT (*itln3*^uta148^) zebrafish at -1 wpi and 2 wpi used for quantifying lymphocyte, myeloid cell and precursor cell populations. FSC = forward scatter, SSC = side scatter. (**C,D**) Lymphocyte fractions of the total cell populations for both WT and *itln3* knockout zebrafish at -1 wpi and 2 wpi. Both the *itln3*^uta145^ (n = 12 in all groups) and *itln3*^uta148^ background fish (n = 8 in all groups) are shown. Blood cell samples were run as technical duplicates. (**E**,**F**) *M. marinum* burden (log10) in 100 ng of zebrafish DNA were measured at 2 wpi and 4 wpi by qPCR in the infected dexamethasone treated zebrafish organ blocks (without the kidney). All bacterial quantification samples were run once. Group sizes at 2 and 4 wpi, respectively, were as follows: WT (*itln3*^*uta145*^) n = 9, n = 8; *iltn3*^uta145/uta145^ n = 9, n = 10; WT (*itln3*^*uta148*^) n = 6, n = 9 and *iltn3*^uta148/uta148^ n = 7, n = 8. A two-tailed Mann-Whitney test was used in the statistical comparison of differences.

We have recently published a zebrafish immune-suppression model for mycobacterial reactivation using orally administered dexamethasone^60^. The dexamethasone treatment decreases the total amount of lymphocytes by an average of 36% (from a relative proportion of 19.3% to 12.4%), and consequently leads to reactivation of the *M. marinum* infection. In turn, a number of studies have suggested that Itln3 functions in microbial surveillance and therefore in the innate immunity^23,24,34^. In order to highlight the importance of innate immune mechanisms in the mycobacterial defense, we used the dexamethasone treatment to specifically deplete the lymphocyte population in the adult *itln3* mutation carrying zebrafish lines *itln3*^uta145^ and *itln3*^uta148^, and subsequently infected both WT and homozygous *itln3* mutants with *M. marinum* (47 CFU; SD 4 CFU) (Fig. 7A). Expectedly, our flow cytometric analysis demonstrated a significant decrease in the lymphocyte counts of both WT *itln3*^uta145^ and *itln3*^uta148^ fish (31.5%, P = 0.002 and 23.7%, P = 0.010, respectively) as well as the *itln3*^uta145/uta145^ and *itln3*^uta148/uta148^ mutants (31.5% and 40.5%, P < 0.001 in both comparisons) three weeks after initiating the dexamethasone administration at 2 wpi (Fig. 7B–D). In addition, neither the total cell count nor the amount of myeloid cells and blood cell precursors were affected by dexamethasone (Supplementary Figure 6). We did not detect any substantial mortality of either the *itln3*^uta145^ or the *itln3*^uta148^ mutants or WT fish during the five-week follow-up period. As is shown in the Fig. 7D,E, the bacterial amounts did not differ between the groups; in 100 ng of zebrafish DNA, mycobacterial copy number medians (log10) of 2.60 and 2.65 in WT *itln3*^uta145^, 2.55 and 3.10 in *itln3*^uta145/uta145^, 2.87 and 2.43 in WT *itln3*^uta148^ and 2.25 and 2.91 in *itln3*^uta148/uta148^ zebrafish were observed at 2 and 4 wpi, respectively.

In conclusion, our data are in accordance with previous literature on the possible role for *itln*s in immunity, as the zebrafish *itln3* is highly induced in a mycobacterial infection. However, *M. marinum* infection experiments using both zebrafish embryos and adult fish suggest that *itln3* is dispensable for a protective mycobacterial host response. Moreover, *itln1* does not seem to compensate for the lack of functional *itln3* in the embryonic infection model. Of note, unlike has been reported for human ITLN1, we were unable to demonstrate direct binding of recombinant Itln3 to mycobacteria (or *S. pneumoniae* or *Escherichia coli*) *in vitro* (Supplementary Figure 7), which may explain the nonessential role of Itln3 for zebrafish immunity in our models.

## Discussion

The genetics of the host affect the outcome of a *M. tuberculosis* infection, i.e. the development of active tuberculosis^4^. Genome-wide expression analyses using microarray and RNA sequencing platforms are important for understanding complicated biological processes such as the host immune defense against pathogens. To date, a handful of transcriptome studies have been done in the zebrafish *M. marinum* infection model using microarray technology^59,72,73^, the digital gene expression (DGE) method^74^ and RNA sequencing^75–77^. Collectively, by using both zebrafish embryos and adult fish, these studies have provided important insights into the innate and adaptive host response against mycobacterial infections.

We used the adult zebrafish *M. marinum* (ATCC 927) infection model together with a zebrafish gene expression microarray to identify novel candidate genes in a mycobacterial infection. From this data, we identified a total of 91 differentially expressed genes (log2 fold change >$|3|$) that were linked to 44 enriched processes, including genes associated with the immune response. Previous studies have shown several genes of the complement system (e.g. *complement component c3b*, *c3b*; *complement component 6*, *c6*) to be up-regulated in an infection^59,72–74,76^, whereas the expression of some complement associated genes (e.g. *complement factor b*, *cfb*; *mannose binding lectin*, *mbl*) has been shown to be reduced^72,74^. In line with previous results, we also saw an induction of *cd59* (regulation of membrane attack complex formation) as well as reduced expression of *cfbl* (component of the C3 convertase). Conversely, although previous transcriptomic studies have shown the induction of genes that are involved in neutrophil and macrophage related functions (e.g. *mpx* and *irg1l*)^73,76^, our data indicated down-regulation of these transcripts in an infection. In summary, the aforementioned similarities and differences between these transcriptomic studies can result from a number of factors including the developmental stage of the host (embryos vs. adult fish), the time points chosen for sample collection, the different outcomes of an infection (chronic vs. acute), the use of different bacterial strains (E11, Mma20 or ATCC 927) and doses, and they can be due to differences in the technical execution of sample preparation and analyses.

Interestingly, circa 38% of the up-regulated probes were related to muscle associated biological processes including muscle contraction (GO:0006936), muscle system process (GO:0003012) and myofibril assembly (GO:0030239). Supporting the relevance of this finding, a genome-wide expression analysis in the fruit fly *Drosophila melanogaster* identified several muscle specific genes such as *actin88F* (A*ct88F*) and *tropomycin 2* (*Tm2*) to be induced after a *Pseudomonas aeruginosa* infection^78^. Consistently, the down-regulation of muscle expressed genes (*troponin C41C, TpnC41C*; *glutathione S-Transferase 2, Gst2*) was later connected to an increased susceptibility to infection, suggesting an immunological role for muscle tissue^79,80^. Although the differential expression of muscle specific genes can be indirectly linked to the immune response through the regulation of other physiological functions, as has been also suggested by Chatterjee *et al*.,^81^, both mouse and zebrafish muscle tissues have also been reported to control the expression of inflammatory cytokine genes (*Tnfa* and *Il6*) upon activation of the immune response^82,83^. In addition, relatively recent studies in the fruit fly and zebrafish have confirmed the importance of immunological signaling pathways in the muscle in both the humoral^81^ and the cellular^80^ immune responses against pathogens. Further studies are required to understand the significance of muscle expressed genes also in the host response against mycobacterial infections.

Lee *et al*.,^21^ described a new carbohydrate-binding lectin family, the Intelectins, with concomitantly proposed function in the innate immunity through microbial recognition^23,34,83^. Two *ITLN* genes (*ITLN1* and *ITLN2*) have been identified in humans, whereas the exact number of protein coding *itln* genes in zebrafish is elusive varying between six (The Zebrafish Information Network, ZFIN) and nine annotated members (Ensembl genome browser). However, not all are expressed in significant amounts. Our genome-wide gene expression analysis showed the highest expression levels for *itln1*, *itln2, itln2-like* and *itln3* in the PBS injected fish with an average log2 expression of 9.6, 11.9, 13.0 and 10.5, respectively (the lowest average log2 expression was 6.9 for a probe A_15_P113269; *ankyrin repeat and kinase domain containing 1*, *ankk1*). This was consistent with Lin *et al*., who reported the highest expression levels for *itln1*, *itln2* and *itln3*^25^. Since their discovery, a large number of studies have reported the induction of the expression of *ITLN* genes in different species in bacterial^25,30–32^, parasite^84–86^ and viral^87^ infections. In addition, although the publicly available transcriptomic data of *M. marinum* infected zebrafish embryos by Benard *et al*., (Gene Expression Omnibus identifier: GSE76499) shows differential regulation of *itln* transcripts in this model^58^, to our knowledge *itln* up-regulation in a mycobacterial infection has not previously been extensively reported in the literature. In our microarray analysis of *M. marinum* infected adult zebrafish, three of the differentially expressed genes were members of the *intelectin* family (*itln2, itln2-like* and *itln3*). The observed decrease in *itln2* expression as well as the induction of *itln3* in these fish was also later confirmed by a qPCR-based quantification of samples from a separate mycobacterial infection experiment. In line with this, a qPCR analysis of *M. marinum* infected zebrafish embryos revealed a significant induction of *itln3* expression post mycobacterial infection, a result which corresponds well with the data by Benard *et al*., (GSE76499)^58^. In embryos, we also observed up-regulation of *itln1*, *itln2* as well as an *itln2-like* in response to a mycobacterial infection. While *itln1* had identical induction kinetics to *itln3* with significant up-regulation starting at 2 dpi, the *itln2-like* and *itln2* genes were induced later in the infection at 4 and 7 dpi, respectively. Of note, the expression of *itln2* at 1 dpi (Fig. 1G) was below the limit of detection in the qPCR. Furthermore, up-regulation of *itln3* was observed after an *S. pneumoniae* infection in zebrafish embryos, suggesting that this gene can be induced also in an immune response against pneumococcus. All in all, in this study we demonstrated the inducibility of *intelectin* genes in both a mycobacterial and a *S. pneumoniae* infection as well as the down-regulation of *itln2* and *itln2-like* transcripts after mycobacterial inoculation in adult zebrafish.

Transcriptomic analyzes have identified several so called classical liver-expressed acute phase protein (APP) genes such as *c-reactive protein* (*crp*) and *serum amyloid a* (*saa*) also in fish species^88–90^. In addition, bacterial infections in rainbow trout (*Oncorhynchus mykiss*)^91^, channel catfish (*Ictalurus punctatus*)^31^ as well as in zebrafish^25^ have resulted in liver-specific induction of certain *itln* gene members. Our mRNA expression analysis of unchallenged zebrafish confirmed the previously published tissue-restricted expression pattern of zebrafish *itln* genes^25^. While *itln2* is expressed almost exclusively in the intestine, the highest relative expression compared to the housekeeping gene was in the liver for *itln3*. In the current study, we produced Strep-tagged® zebrafish Itln3 in a mammalian expression system to study whether Itln3 could act as a potential APP in microbial recognition. Similarly to human ITLN1^34,83,92^, zebrafish Itln3 was secreted into the culture media. However, although Tsuji *et al*., (2009) has reported the ability of human ITLN1 to bind to galactofuranosyl (Galf) residues on the mycobacterial cell membrane^83^, recombinant Itln3 did not bind readily to *M. marinum in vitro*. Similarly, Itln3 did not bind to *E. coli* or *S. pneumoniae* in our hands.

To our knowledge, only two *in vivo* studies on the significance of Intelectins in the host response against pathogens have been conducted^35,36^. While the lack of ITLN2 was associated with an increased susceptibility toward the parasite *T. spiralis* in C57BL/10 mice^36^, the over-expression of ITLN1 and ITLN2 in the lungs of transgenic mice could not restrict either a *Nippostrongylus brasiliensis* or a *Mycobacterium tuberculosis* infection differently from the littermate controls^35^. While genes of the innate immune system can be studied autonomously in zebrafish embryos, which lack a functional adaptive immunity^50^, adult zebrafish have a highly similar immune system compared to humans^93^. Correspondingly, the embryonic *M. marinum* infection model has revealed important insights into the mechanisms of the innate immunity in mycobacterial host resistance (reviewed in^50,51^) and the adult model has proven its usefulness e.g. in modelling a latent infection^56^ and disease reactivation^56,60^. In order to obtain a more comprehensive view about the *in vivo* significance of ITLNs in a mycobacterial infection, we used the *itln3* deficient zebrafish together with both the embryonic as well as the adult *M. marinum* models. In these experiments, the comparison of survival and mycobacterial burden between *itln3* mutant fish and their WT siblings did not reveal any differences in either of the mutant lines (*itln3*^uta145^ and *itln3*^uta148^). Also, it was demonstrated that this was independent of the site or timing of the microinjection in the embryos (yolk sac at 0 dpf vs. blood circulation valley at 2 dpf). Collectively, we conclude that zebrafish *itln3* is not required for the resistance against a mycobacterial infection.

Genetic compensation is a well-known phenomenon in model organisms with experimental gene knockouts^70^. In this process, the specific function of a knockout gene can be restored by additional naturally occurring mutations or transcriptional changes in other genes^70^. Here, we report the up-regulation of *itln3* as well as another member of the *intelectin* family, *itln1*, in a *M. marinum* infection of zebrafish embryos with analogous induction kinetics. To overcome potential compensatory effects of *itln1* in our *itln3* mutants, we knocked down *itln1* by morpholinos and performed simultaneous *M. marinum* infections in the *itln3* mutant embryos. Silencing *itln1* in *itln3* mutants during a *M. marinum* infection did not reveal any differences compared to controls, demonstrating that *itln1* expression does not compensate for the lack of a functional *itln3* in a *M. marinum* infection. Moreover, our qPCR quantification of *itln1*, *itln2* and *itln2-like* in the uninjected and *M. marinum* infected zebrafish embryos as well as adult zebrafish did not reveal transcriptional compensation for *itln3* in the homozygous mutant background. Similarly, the depletion of lymphocytes in the adult zebrafish did not reveal the importance for Itln3 in the immunity against a *M. marinum* infection. All in all, our data indicate that despite being strongly induced by a mycobacterial infection, *itln3* is dispensable for the immune response against *M. marinum* both in embryonic and adult zebrafish.

## Methods

### Zebrafish lines and maintenance

The zebrafish maintenance and all of the experiments were in accordance with the Finnish Act on the Protection of Animals Used for Scientific or Educational Purposes (497/2013) as well as the EU Directive on the Protection of Animals Used for Scientific Purposes (2010/63/EU). Experiments were approved by the Animal Experiment Board of Finland (permit for zebrafish maintenance: ESAVI/10079/04.10.06/2015; permits for the experiments: ESAVI/2464/04.10.07/2017, ESAVI/10823/04.10.07/2016, ESAVI/2235/04.10.07/2015 and ESAVI/11133/04.10.07/2017). WT AB fish as well as in-house CRISPR/Cas9 produced F2-generation *itln3*^uta145^ and F2- or F3-generation *itln3*^uta148^ mutant zebrafish were used in the embryonic experiments, whereas three- to seven-month-old AB, *il10*^e46^, *itln3*^uta145^ and *itln3*^uta148^ zebrafish were used in the experiments with the adult fish. Zebrafish embryos were maintained according to standard protocols in embryonic medium E3 (5 mM NaCl, 0.17 mM KCl, 0.33 mM CaCl_2_, 0.33 mM MgSO_4_, 0.0003 g/l methylene blue) at 28.5 °C until 7 dpf. Maintenance of the adult zebrafish was as follows; unchallenged fish were kept in a conventional flow through system (Aquatic Habitats, Florida, USA) with an automated light/dark cycle of 14 h/10 h and fed once a day with Gemma Micro 500 (Skretting, Stavanger, Norway) or twice with SDS 400 (Special Diets Services, Essex, UK) feed. *M. marinum* infected adults were kept in a separate flow through system (Aqua Schwarz GMbH, Göttingen, Germany) with the above-mentioned light/dark cycle and fed once a day with Gemma Micro 500 (Skretting) or SDS 400 (Special Diets Services). Infected fish were monitored daily. Humane endpoint criteria pre-defined in the animal experiment permits were applied throughout the follow-up.

### Experimental *M. marinum* infections

*M. marinum* (ATCC 927 -strain) culture and the adult zebrafish inoculations were performed as described previously^56^. In the *M. marinum* infections of the zebrafish embryos, a total volume of 1–2 nl was microinjected either into the yolk sac (0 dpf) or into the blood circulation valley (2 dpf) by using a borosilicate capillary needle (Sutter instrument Co., California, USA), a micromanipulator (Narishige International, London UK) and a PV830 Pneumatic PicoPump (World Precision Instruments, Florida, USA). 10 mM phosphate buffered saline (PBS) with 2% polyvinylpyrrolidone-40 (PVP) (Sigma-Aldrich) and 0.3 mg/ml phenol red (Sigma-Aldrich) was used as a mycobacterial carrier solution. Prior to circulation valley injections, the 2 dpf zebrafish were anesthetized with 0.02% 3-amino benzoic acid ethyl ester (Sigma-Aldrich). Embryonic infections were visualized with a Stemi 2000 microscope (Carl Zeiss MicroImaging GmbH, Göttingen, Germany) and the survival of the embryos followed daily. Adult zebrafish were first anesthetized with 0.02% 3-amino benzoic acid ethyl ester (Sigma-Aldrich, Missouri, USA), and then injected with 5 µl of *M. marinum* in a suspension of 10 mM PBS and 0.3 mg/ml phenol red (Sigma-Aldrich, Missouri, USA) into the abdominal cavity using a 30 gauge Omnican 100 insulin needle (Braun, Melsungen, Germany). The *M. marinum* amounts from both the embryonic and adult infections were verified by plating bacterial inoculates on 7H10 agar (Becton Dickinson, New Jersey, USA) plates and counting the colony forming units (CFU) 5-days after plating.

### Gene expression microarray

RNA was extracted from the zebrafish organ blocks (includes all the organs of the abdominal cavity) with TRIreagent (Molecular Research Center, Ohio, USA) following the manufacturer’s protocol. Microarray procedures were carried out by the Turku Centre for Biotechnology at the Finnish Microarray and Sequencing Centre by using a Zebrafish (V3) Gene Expression Microarray, 4 × 44 K (Agilent Technologies, California, USA). In short, 100 ng of total RNA was amplified and Cy3-labeled with Low Input Quick Amp Labeling kit, one-color (Agilent), processed using the RNA Spike-In Kit, one-color (Agilent) and quality controlled with 2100 bioanalyzer RNA 6000 Nano kit (Agilent). Labelling and hybridization of the transcripts were done onto Agilent’s 4 × 44 K Zebrafish v3 array (Design ID 026437) using GE Hybridization Kit (Agilent). Microarrays were scanned with an Agilent scanner G2565CA using a profile AgilentHD_GX_1Color. Numerical results were obtained with Feature Extraction Software v. 10.7.3 (Agilent) with the protocol GE1_107_Sep09 and the signal intensities normalized prior further analysis. Cut-off value (log2 fold change >$|3|$) for the up- and down-regulated genes was chosen in order to obtain approximately 100 differentially expressed candidate genes for further evaluation. Gene ontology enrichment analysis was performed using GOrilla^61,62^ with two unranked lists of genes (Target list: log2 fold change >$|3|$, Background list: log2 fold change <$|3|$) using *Danio rerio* genome assembly.

### qPCR

For gene expression analysis of the zebrafish embryo samples, genomic DNA (gDNA) removal and RNA isolation were performed using the RNeasy Plus Mini Kit (Qiagen, Hilden, Germany) according to the manufacturer’s guidelines. Adult zebrafish RNA was extracted from the organ blocks, liver, spleen, kidney and intestine with TRIreagent (Molecular Research Center) following the associated protocol. The genomic DNA (gDNA) from the RNA samples of the adult fish was removed with RapidOut DNA Removal Kit (Thermo Fischer Scientific, Waltham, USA). RNA quality was controlled with either 1.5% agarose Tris-acetate-EDTA (TAE) gel electrophoresis or by using Fragment Analyzer system (Advanced Analytical, Inc., Ankeny, USA) and the Standard Sensitivity RNA Analysis Kit (15 nt) (Advanced Analytical). All reverse transcriptions were done by using the SensiFAST^TM^ cDNA synthesis kit (BioLine, London, UK), and the gene expression levels of the target genes were determined from the cDNA with quantitative PCR (qPCR) using the PowerUp™ SYBR® master mix (Thermo Fischer Scientific) and CFX96™ detection system (Bio-Rad Laboratories, California, USA). CFX Manager software (v. 3.1; Bio-Rad Laboratories) was used for data analysis. Target gene expression was normalized to the *eukaryotic translation elongation factor 1 alpha 1, like 1* (*eef1a1l1 or ef1a*)^94^ expression using the 2^−ΔCt^ method. *M. marinum* burden from the zebrafish was determined from the total DNA by qPCR with *MMITS*-specific primers^56^. Embryo DNA for mycobacterial quantification was isolated with standard ethanol precipitation procedure utilizing the following lysis buffer: 10 mM Tris (pH 8.2), 10 mM EDTA, 200 mM NaCl, 0.5% SDS, 200 µg/ml Proteinase K (Thermo Fischer Scientific), whereas TRIreagent (Molecular Research Center) was used for the adult fish DNA isolations. No reverse transcriptase control samples were added to the gene expression analyses, and no template control (H_2_O) samples were included in all of the qPCR experiments to preclude contamination. Specificity and the correct size of the qPCR products were verified by melt curve analysis and 1.5% agarose TAE gel electrophoresis. Undetectable qPCR products with incorrect melt curves were given a Ct-value of 40 for the gene expression analyses, and the expression was considered to be below detection. qPCR primers used in the study are listed in Supplementary Table 3.

### CRISPR/Cas9 mutagenesis

We have previously set-up our in-house zebrafish CRISPR/Cas9 mutagenesis method based on the protocol published by Hruscha and Schmid (2015)^65,95^. First, guide RNA (gRNA) target sequences for *itln3* were designed with the CRISPR design tool (http://crispr.mit.edu/), and validated with the Casellas laboratory sgRNA tool^96^ and the standard nucleotide BLAST analysis^97^. *itln3* exon 2 gRNA was produced by *in vitro* transcription using the MEGAshortscript T7 Transcription Kit (Ambion Life Technologies, CA, USA). 2000 pg of gRNA, 330 pg of *cas9* mRNA (Sigma-Aldrich and Invitrogen, California, USA) and 1.5 ng of phenol red (Sigma-Aldrich) tracer were injected into one-cell-stage AB zebrafish embryos, and the success of mutagenesis was evaluated with the T7 endonuclease I (T7EI)- and the heteroduplex mobility assay (HMA) from isolated DNA of 2 dpf embryos^49^. Gel images were obtained with ChemiDoc™ XRS+ system (Bio-Rad Laboratories) and analyzed with Image Lab software (v. 5.2; Bio-Rad Laboratories). To establish the *itln3* knockout fish line, gRNA was microinjected into zebrafish embryos and the F0-generation fish grown to adulthood. Individual outcrosses of the F0-zebrafish with the Tupfel long fin (TL) fish allowed us to screen for germline transmitted mutations and to identify nonsense mutations of interest in the F1-progeny. The F1-progeny screen was done from the tailfin DNA of the adult zebrafish with HMA followed by Sanger sequencing in our institutes core-facility (MED, University of Tampere). The F1-zebrafish carrying individual mutations of interest were spawn together to obtain F2-generation progeny for the experiments. In the end, a total of two different *itln3* mutation (*itln3*^uta145^ and *itln3*^uta148^) bearing zebrafish lines were used in the study.

### PCR based genotyping

F2-generation *itln3*^uta145^ and *itln3*^uta148^ zebrafish lines were mainly genotyped using PCR. To this end, template DNA was either isolated using a standard ethanol precipitation protocol, or with a rapid tissue lysis protocol^98^. Primers were designed for both the WT and the mutated sequences at the gRNA target region; WT *itln3*^uta145^ F: 5′-ATGCTAGGTTGAGGAGCATC-3′, mutant *itln3*^uta145^ F: 5′-ATGCTAGGTTGAGGAGCTCG-3′, WT *itln3*^uta148^ F: 5′-CTAGGTTGAGGAGCATCGCT-3′, mutant *itln3*^uta148^ R: 5′-CCGAGCTGATACTTACCTAGC-3′, and amplified with the appropriate flanking primer: F: 5′-GGAGCTGTCACTCCAAAGCC-3′ or R: 5′-GTGGTTGATCAACCATTCAGCAC-3′. To determine the genotypes of the *itln3*^uta145^ and *itln3*^uta148^ zebrafish, individual PCR reactions with both WT and mutant primer pairs were prepared for each fish and 1.5% agarose TAE gel electrophoresis was performed to analyze the PCR products.

### Morpholino injections

Splice-blocking morpholino (SB) for *itln1* (5′-CTAATTCTGTACTTACTCGATTCAC-3′) was designed by and ordered from GeneTools, LLC (Philomath, Oregon, USA). The targeted genomic sequence was verified from our AB and *itln3* knockout zebrafish lines by sequencing^99^. In order to ensure no adverse effects on survival or the phenotype of the morpholino injected embryos in later experiments, the oligonucleotide dosage was first titrated by using three different quantities (7.1 ng, 2.8 ng and 1.1 ng), and the survival of the embryos was observed daily until 7 dpf. The embryos were imaged using Zeiss Lumar V12 fluorescence microscope. The selected microinjection volume was set to 2 nl containing 2.8 ng of SB or random control (RC) morpholino as well as 7 mg/ml of tetramethylrhodamine dextran (Thermo Fisher Scientific) or 0.3 mg/ml phenol red tracer suspended in PBS. In the morpholino-*M. marinum* co-injections, the previously described suspension with 2% PVP was used as a mycobacterial carrier solution. All of the morpholino injections as well as morpholino and *M. marinum* co-injections were done before the 16-cell-stage of development into the yolk sac of the embryos. Similarly than in the other *M. marinum* infection experiments, the mycobacterial counts in the injections were verified by plating. Primers used for the morpholino target site sequencing were F: 5′-TGCACAGGTATTCACCATTTTATGATG-3′ and R: 5′-AAGTTCTCTGCAGCTTCTTGC-3′ and for the verification of the morpholino functionality as well as quantification of the WT *itln1* expression by qPCR: F: 5′-ATGATGCAGTCAGCTGGTTTTCTTCTG-3′ and R: 5′-GCAGTGACCGACTCTGGAAATTCTCC-3′.

### Flow cytometry

Flow cytometry for the adult zebrafish kidney cells was performed as described previously^71^. Briefly, *itln3*^uta145^ and *itln3*^uta148^ fish were euthanized with 0.04% 3-amino benzoic acid ethyl ester and their kidneys isolated and suspended in PBS supplemented with 0.5% fetal bovine serum (Sigma-Aldrich). Prior analysis, the kidney cells were filtered through a cell strainer cap with a 35 µm mesh (Corning/Thermo Fisher Scientific). Relative amounts of lymphocytes, myeloid cells and blood cell precursors were determined with a FACSCanto II instrument (Becton, Dickinson, New Jersey, USA) and the data was analyzed with the FlowJo program (v. 7.5; Tree Star, Inc, Oregon, USA). Gating of the blood cell populations is based on previous publications^60,71,100,101^.

### Dexamethasone mediated immunosuppression

Similarly as described previously^60^, 25 mg of dexamethasone (Sigma-Aldrich) was mixed with gelatin (Sigma-Aldrich) and used to coat 10 g of SDS400 food (Special Diets Services). During the experiment, a daily dose of 10 µg of dexamethasone (4 mg of food) was given per fish for a total of 5 weeks. A new batch of dexamethasone food was prepared for usage every second week. Dexamethasone was administered for a total of five weeks and the well-being of the fish monitored daily.

### Statistical analysis

Sample size calculations have been described in our previous publication^100^. Statistical analyses were done with the Prism v. 5.02 (GraphPad Software, California, USA). In the survival experiments a log-rank (Mantel-Cox) test was used, whereas in the flow cytometry and qPCR analyses a nonparametric two-tailed Mann-Whitney analysis was performed. *P* values of < 0.05 were considered significant.

## Supplementary information

Supplementary information

## Data Availability

Gene expression microarray data has been submitted to Gene Expression Omnibus (GEO) repository and can be found with the identifier code: GSE120552. Other generated and analyzed data is available on reasonable request from the corresponding author.
