# Supplementary material for: Intelectin 3 is dispensable for resistance against a mycobacterial infection in zebrafish (Danio rerio)
Source: Sci Rep. 2019 Jan 30;9:995. doi: 10.1038/s41598-018-37678-1 (PMC6353920; doi:10.1038/s41598-018-37678-1)
Supplement: Supplementary file 1 — Supplementary information [file 41598_2018_37678_MOESM1_ESM.pdf]

Supplementary information

Intelectin 3 is dispensable for resistance against a mycobacterial infection in zebrafish (*Danio rerio*)

Markus J.T. Ojanen[1,2], Meri I.E. Uusi-Mäkelä[1], Sanna-Kaisa E. Harjula[1], Anni K. Saralahti[1], Kaisa E. Oksanen[1], Niklas Kähkönen[3], Juha A.E. Määttä[3], Vesa P. Hytönen[3], Marko Pesu[2,4], Mika Rämet[*,1,5,6,7]

Affiliations:

[1]Laboratory of Experimental Immunology, BioMediTech Institute and Faculty of Medicine and Life Sciences, University of Tampere, Tampere, Finland;

[2]Laboratory of Immunoregulation, BioMediTech Institute and Faculty of Medicine and Life Sciences, University of Tampere, Tampere, Finland;

[3]Laboratory of Protein Dynamics, BioMediTech Institute and Faculty of Medicine and Life Sciences, University of Tampere, Tampere, Finland;

[4]Department of Dermatology, Tampere University Hospital, Tampere, Finland;

[5]Department of Pediatrics, Tampere University Hospital, Tampere, Finland;

[6]Department of Children and Adolescents, Oulu University Hospital, Oulu, Finland;

[7]PEDEGO Research Unit and Medical Research Center Oulu, University of Oulu, Oulu, Finland

***Corresponding author:** Correspondence to Mika Rämet, phone: 358-50-4336276, Email: mika.ramet@uta.fi

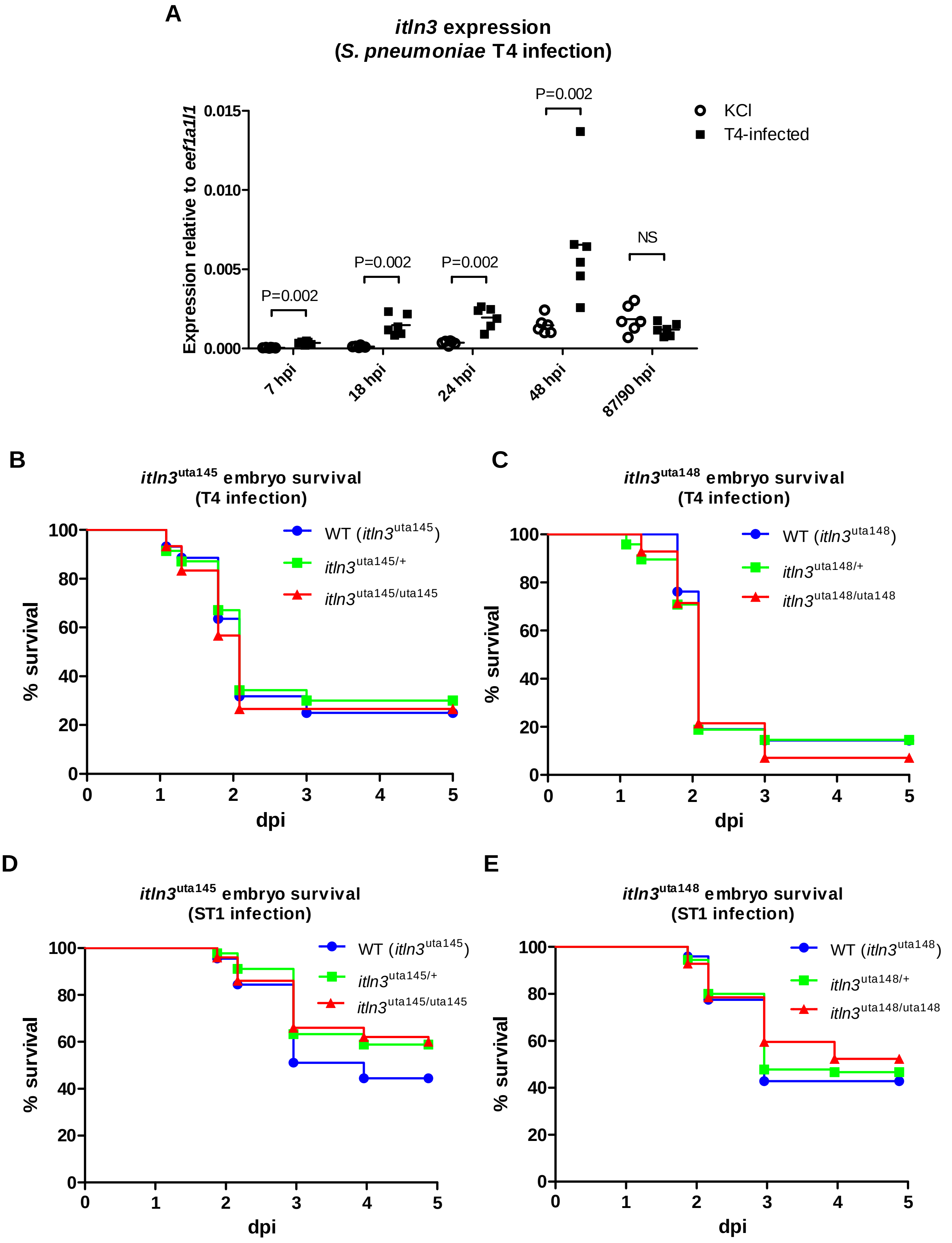
A
itln3 expression
(S. pneumoniae T4 infection)
Expression relative to eef1a1l1
0.015
0.010
0.005
0.000
P=0.002
P=0.002
P=0.002
P=0.002
NS
KCl
T4-infected
7 hpi
18 hpi
24 hpi
48 hpi
87/90 hpi
B
itln3uta145 embryo survival
(T4 infection)
WT (itln3uta145)
itln3uta145/+
itln3uta145/uta145
% survival
dpi
C
itln3uta148 embryo survival
(T4 infection)
WT (itln3uta148)
itln3uta148/+
itln3uta148/uta148
% survival
dpi
D
itln3uta145 embryo survival
(ST1 infection)
WT (itln3uta145)
itln3uta145/+
itln3uta145/uta145
% survival
dpi
E
itln3uta148 embryo survival
(ST1 infection)
WT (itln3uta148)
itln3uta148/+
itln3uta148/uta148
% survival
dpi

Supplementary Figure 1. *itln3* expression is induced in *S. pneumoniae* infected zebrafish embryos, but *itln3* is dispensable for embryo survival. A) The expression of *itln3* was measured with qPCR from zebrafish embryos infected with *S. pneumoniae* (serotype T4; 296 CFU, SD 32 CFU) 7 hpi to 90 hpi, and compared to potassium chloride (KCl) injected control fish (n=6 in both groups at all timepoints). Gene expressions were normalized to *eef1a1l1* expression. All samples were run once. A two-tailed Mann-Whitney test was used in the statistical comparison of differences. **B-C)** Both *itln3*^{uta145} (n=31-70) and *itln3*^{uta145} (n=14-48) background zebrafish embryos were infected with *S. pneumoniae* (serotype T4; 380 CFU, SD 172 CFU) and their survival followed until 5 dpi. **D-E)** The *itln3*^{uta145} (n=45-91) and *itln3*^{uta145} background (n=42-91) zebrafish embryos were infected with *S. pneumoniae* (serotype 1; 48 CFU, SD 28 CFU) and their survival followed until 5 dpi. The data was collected from a single experiment in panels B-E and a log-rank (Mantel-Cox) test was used for the statistical comparison of differences.

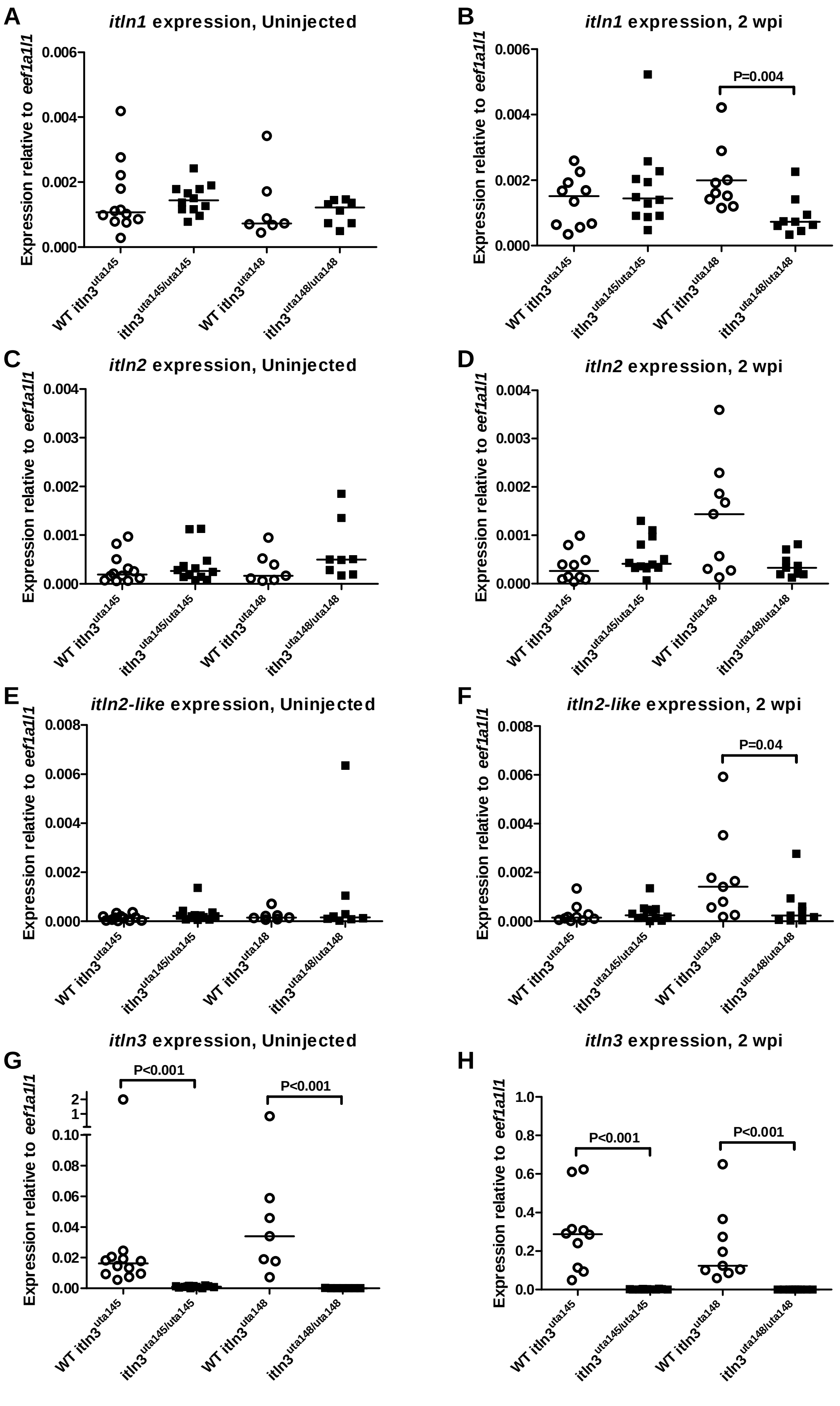

A
itln1 expression, Uninjected
B
itln1 expression, 2 wpi
P=0.004
C
itln2 expression, Uninjected
D
itln2 expression, 2 wpi
E
itln2-like expression, Uninjected
F
itln2-like expression, 2 wpi
P=0.04
G
itln3 expression, Uninjected
P<0.001
P<0.001
H
itln3 expression, 2 wpi
P<0.001
P<0.001
Expression relative to eef1a1l1
WT itln3uta145
itln3uta145/uta145
WT itln3uta148
itln3uta148/uta148

Supplementary Figure 2. Expression of zebrafish *itln* genes in uninjected and *M. marinum* infected adult *itln3*^{uta145} and *itln3*^{uta148} zebrafish. The expression of zebrafish *itln* genes (*itln1*, *itln2*, *itln2-like* and *itln3*) was measured with qPCR in the organ blocks (without the kidney) of uninjected and *M. marinum* infected (422 CFU; SD 221 CFU, 2 wpi) adult WT (*itln3*^{uta145}) (n=12 and n=10, respectively), *itln3*$^{uta145/145}$(n=12 and n=12, respectively), WT (*itln3*^{uta148}) (n=7 and n=9, respectively) and *itln3*$^{uta148/148}$ zebrafish (n=7-8 and n=9-10, respectively). Note the different scales of the y axes and the divided axis in panel G. Gene expressions were normalized to *eef1a1l1* expression. All samples were run once. A two-tailed Mann-Whitney test was used in the statistical comparison of differences.

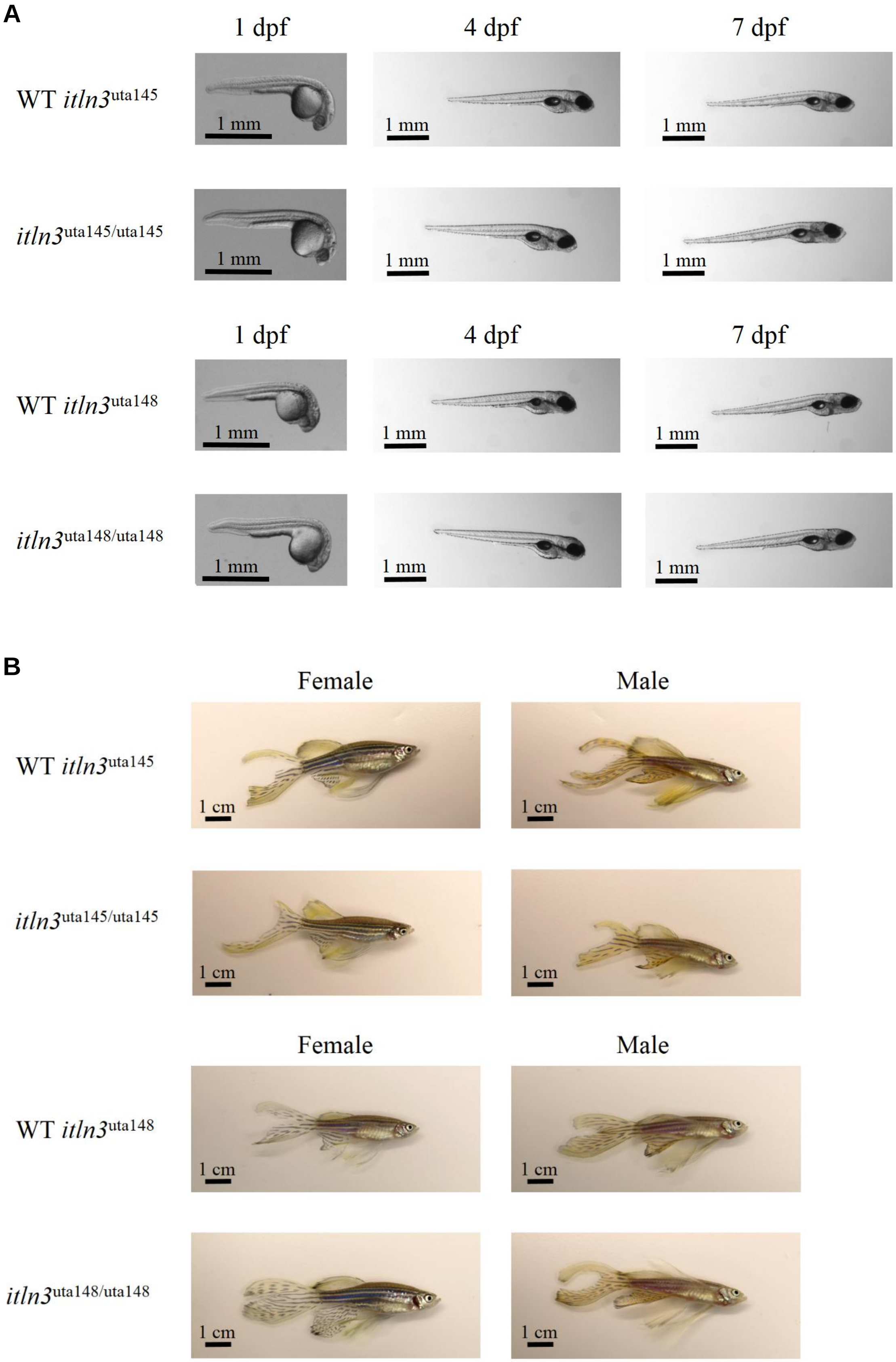
A
1 dpf
4 dpf
7 dpf
WT itln3uta145
itln3uta145/uta145
1 dpf
4 dpf
7 dpf
WT itln3uta148
itln3uta148/uta148
1 mm
B
Female
Male
WT itln3uta145
itln3uta145/uta145
Female
Male
WT itln3uta148
itln3uta148/uta148
1 cm

Supplementary Figure 3. Homozygous *itln3*$^{\text{uta145/uta145}}$ and *itln3*$^{\text{uta148/uta148}}$ mutants develop normally. A) F3-progeny of *itln3*$^{\text{uta145/+}}$ and *itln3*$^{uta148/+}$ zebrafish were imaged at 1, 4 and 7 dpf. The larvae were anesthetized for imaging with 0.02% 3-amino benzoic acid ethyl ester (Sigma-Aldrich) at 4 and 7 dpf, and the embryos collected for genotyping at 7 dpf. Representative images of *itln3*$^{\text{uta145/145}}$ and *itln3*$^{\text{uta148/148}}$ mutants as well as the corresponding WT embryos are shown. Micrographs were taken with Zeiss Lumar V12 fluorescence microscope and Axiocam MRm digital camera using a bright field exposure of 2 ms. A 23.5x-magnification was used at 1 dpf and a 15.0x-magnification at 4 and 7 dpf. **B)** 12-month-old WT (*itln3*$^{\text{uta145}}$), *itln3*$^{\text{uta145/145}}$, WT (*itln3*$^{\text{uta148}}$) and *itln3*$^{\text{uta148/148}}$ female and male zebrafish were anesthetized with 0.02% 3-amino benzoic acid ethyl ester (Sigma-Aldrich) and imaged submerged in water using Canon EOS 7D Mark II camera with an exposure time of 8 ms. Brightness was increased by 20% for all of the images in panel B using Windows Photo Viewer. All images in panels A-B were cropped to exclude unnecessary empty background from the figure.

A

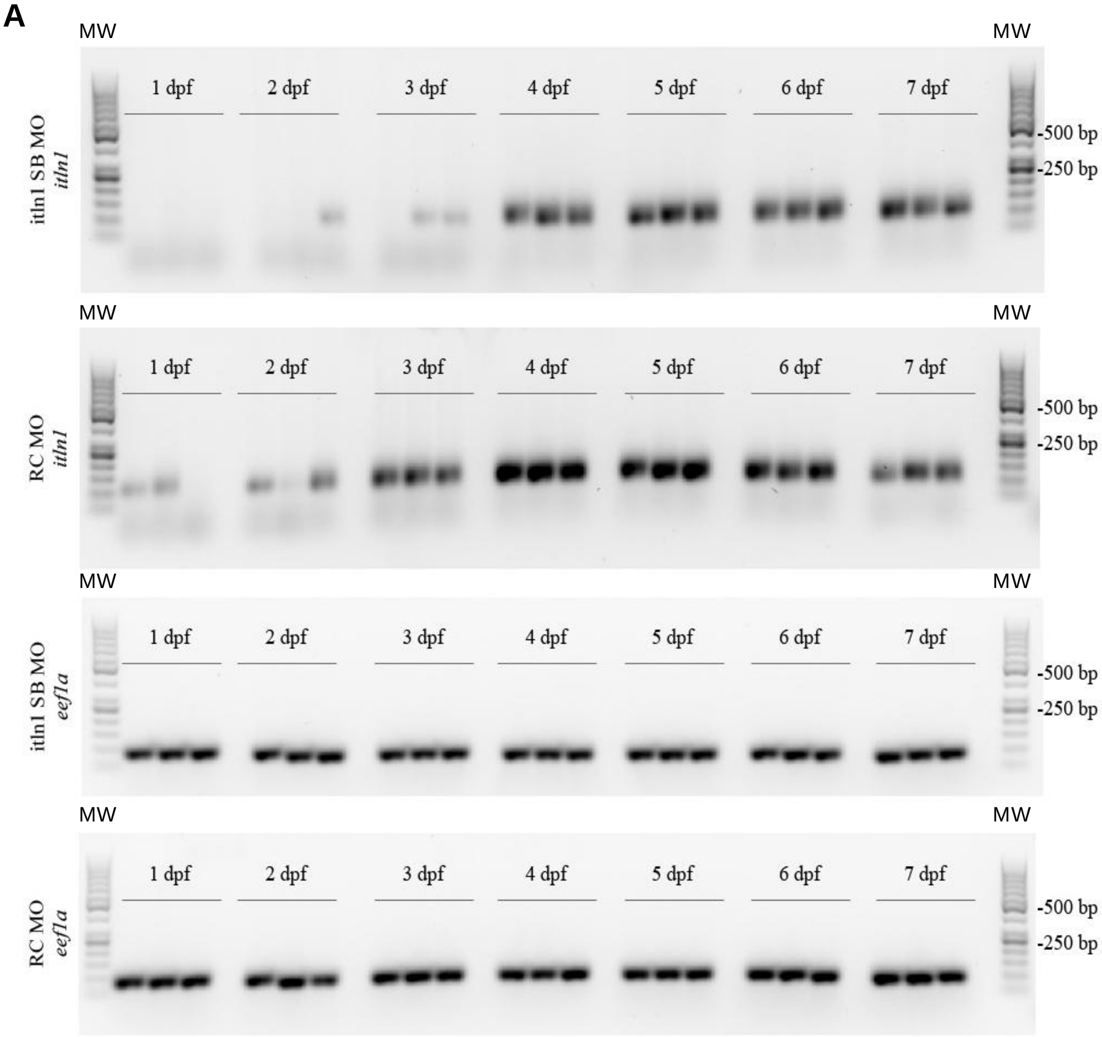

MW
MW
1 dpf
2 dpf
3 dpf
4 dpf
5 dpf
6 dpf
7 dpf
-500 bp
-250 bp
itln1 SB MO
itln1
RC MO
itln1
itln1 SB MO
eef1a
RC MO
eef1a

B

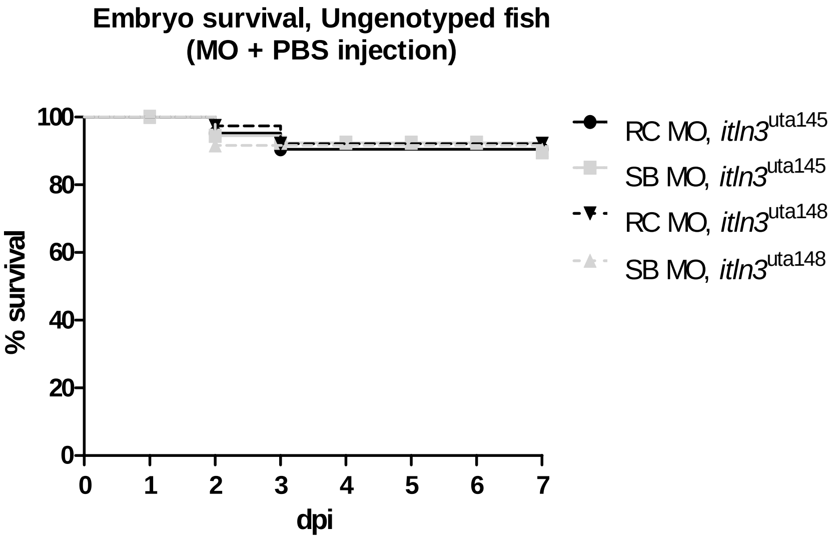

Embryo survival, Ungenotyped fish
(MO + PBS injection)
% survival
dpi
0
20
40
60
80
100
0
1
2
3
4
5
6
7
RC MO, $itln3^{uta145}$
SB MO, $itln3^{uta145}$
RC MO, $itln3^{uta148}$
SB MO, $itln3^{uta148}$

Supplementary Figure 4. ***Itln1* splice site blocking morpholino knocks down the expression of *itln1*, but does not alter the survival of the embryos. A)** The expression of *itln1* was measured with qPCR in the splice blocking morpholino (SB MO) (n=3 at all timepoints) and the random control morpholino (RC MO) (n=3 at all timepoints) injected WT AB zebrafish embryos between 0 dpf and 7 dpf (See Figure 5C). The qPCR amplified samples were run with 1.5% agarose TAE gel electrophoresis to confirm knockdown effects and to compare the intensities of the *itln1* SB MO and RC MO injected samples. GeneRuler 50 bp DNA Ladder (#SM0373, Thermo Fischer Scientific) was used as a molecular weight marker (MW). The housekeeping gene *eef1a1l1* from the same samples was also amplified and analyzed with gel electrophoresis. Images were obtained with ChemiDoc™ XRS+ system (Bio-Rad Laboratories) and analyzed with Image Lab software (v. 5.2; Bio-Rad Laboratories). Gel images are cropped to exclude portions that do not contain experimental samples. **B)** Survival of the morpholino and phosphate buffered saline (PBS) co-injected (yolk sac injection) ungenotyped embryos from both the $itln3^{uta145}$ (RC, n=42; SB n=58) and $itln3^{uta148}$ (RC, n=38; SB, n=72) background were followed until 7 dpi. The data was collected from a single experiment. A log-rank (Mantel-Cox) test was used for the statistical comparison of differences. RC= random control, SB= slice blocking, MO = morpholino.

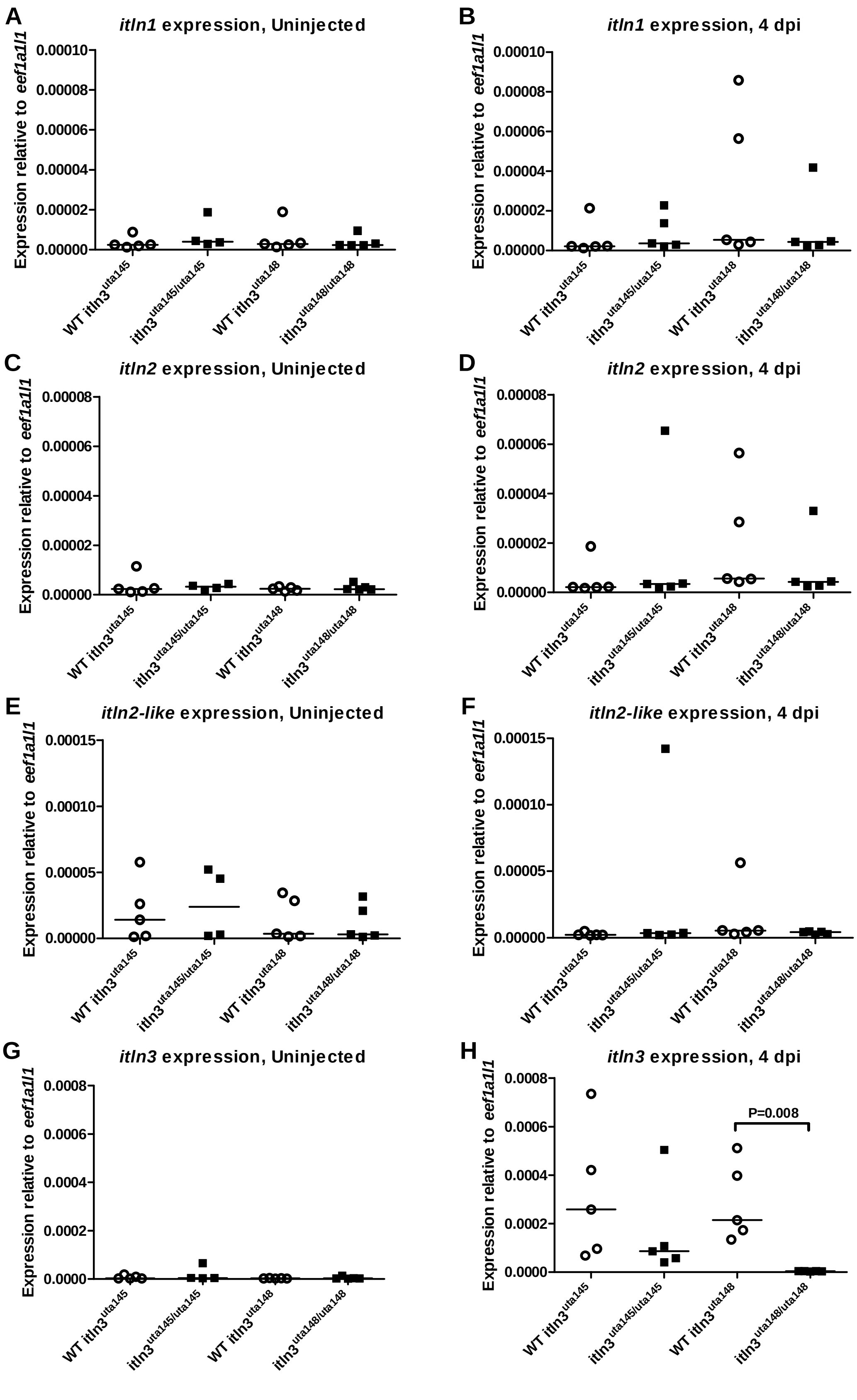
A
itln1 expression, Uninjected
Expression relative to eef1a1l1
0.00010
0.00008
0.00006
0.00004
0.00002
0.00000
WT itln3uta145
itln3uta145/uta145
WT itln3uta148
itln3uta148/uta148
B
itln1 expression, 4 dpi
C
itln2 expression, Uninjected
0.00008
0.00006
0.00004
0.00002
0.00000
D
itln2 expression, 4 dpi
E
itln2-like expression, Uninjected
0.00015
0.00010
0.00005
0.00000
F
itln2-like expression, 4 dpi
G
itln3 expression, Uninjected
0.0008
0.0006
0.0004
0.0002
0.0000
H
itln3 expression, 4 dpi
P=0.008

Supplementary Figure 5. Expression of zebrafish *itln* genes in uninjected and *M. marinum* infected *itln3*^{uta145} and *itln3*^{uta148} zebrafish embryos. The expression of zebrafish *itln* genes (*itln1*, *itln2*, *itln2-like* and *itln3*) was measured with qPCR in the uninjected (4 dpf) and *M. marinum* infected (25 CFU; SD 23 CFU, 4 dpf/ 4 dpi) WT (*itln3*^{uta145}) (n=5 in both groups), *itln3*$^{uta145/145}$(n=4 and n=5, respectively), WT (*itln3*^{uta148}) (n=5 in both groups) and *itln3*$^{uta148/148}$ zebrafish embryos (n=5 in both groups). Individual embryos were used in the *itln3*^{uta145} background samples, whereas *itln3*^{uta148} background samples were pooled from 4 to 6 embryos. Note the different scales of the y axes. Gene expressions were normalized to *eef1a1l1* expression. Target genes were run once. A two-tailed Mann-Whitney test was used in the statistical comparison of differences.

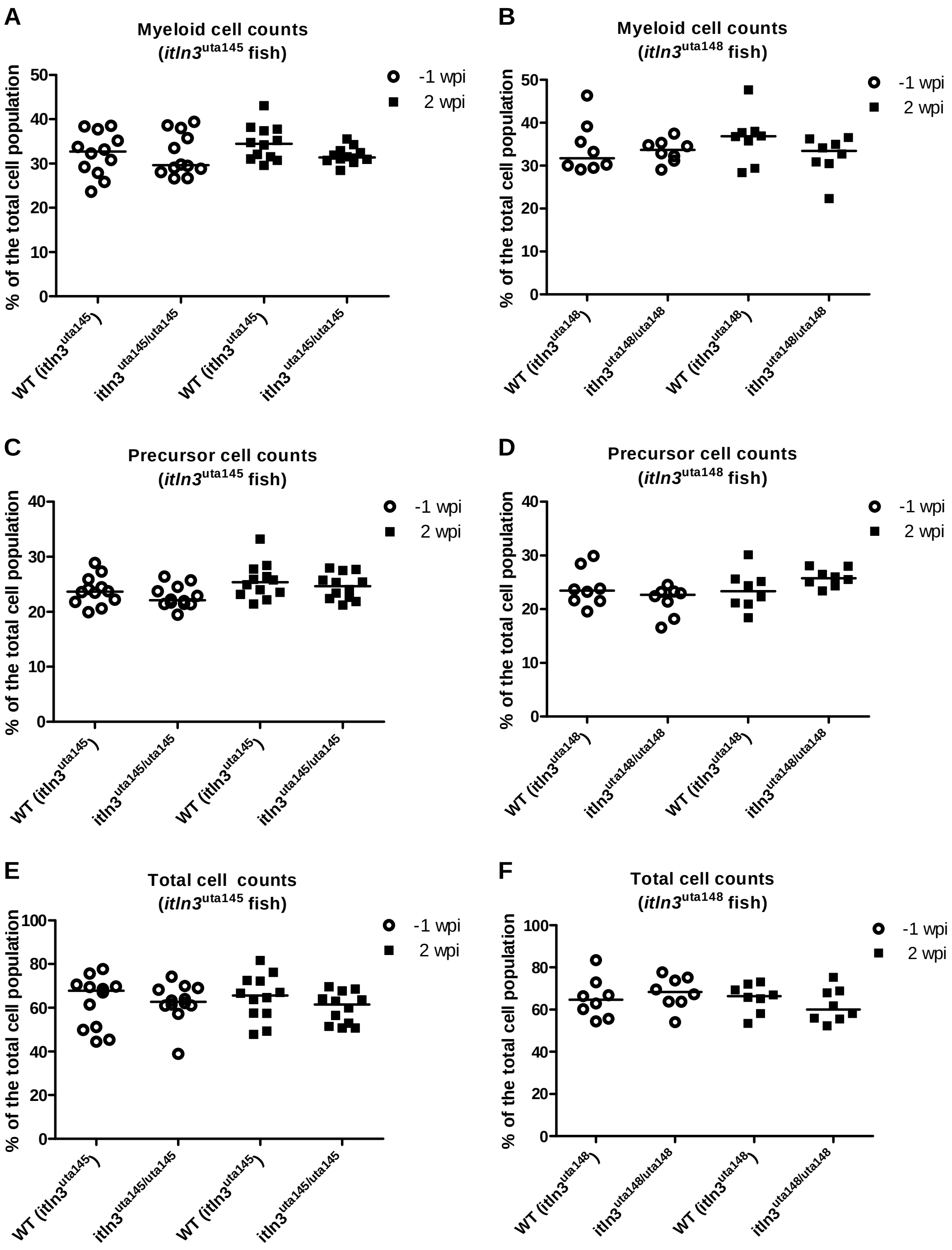

Supplementary Figure 6. Quantification of myeloid cells, precursor cells and the total cell count in the dexamethasone treated zebrafish. A-B) The fraction of myeloid cells from the total cell population for the WT (*itln3*^{uta145}) and *itln3*$^{uta145/uta145}$ as well as WT (*itln3*^{uta148}) and *itln3*$^{uta148/uta148}$ were measured with flow cytometry at -1 wpi and 2 wpi (n=12 for all of the *itln3*^{uta145} fish groups and n=8 for all of the *itln3*^{uta148} groups). Flow cytometry was used to determine also the **C-D)** precursor cell and **E-F)** the total cell counts from the same samples. Technical duplicates were run from all of the samples. A two-tailed Mann-Whitney test was used in the statistical comparison of differences.

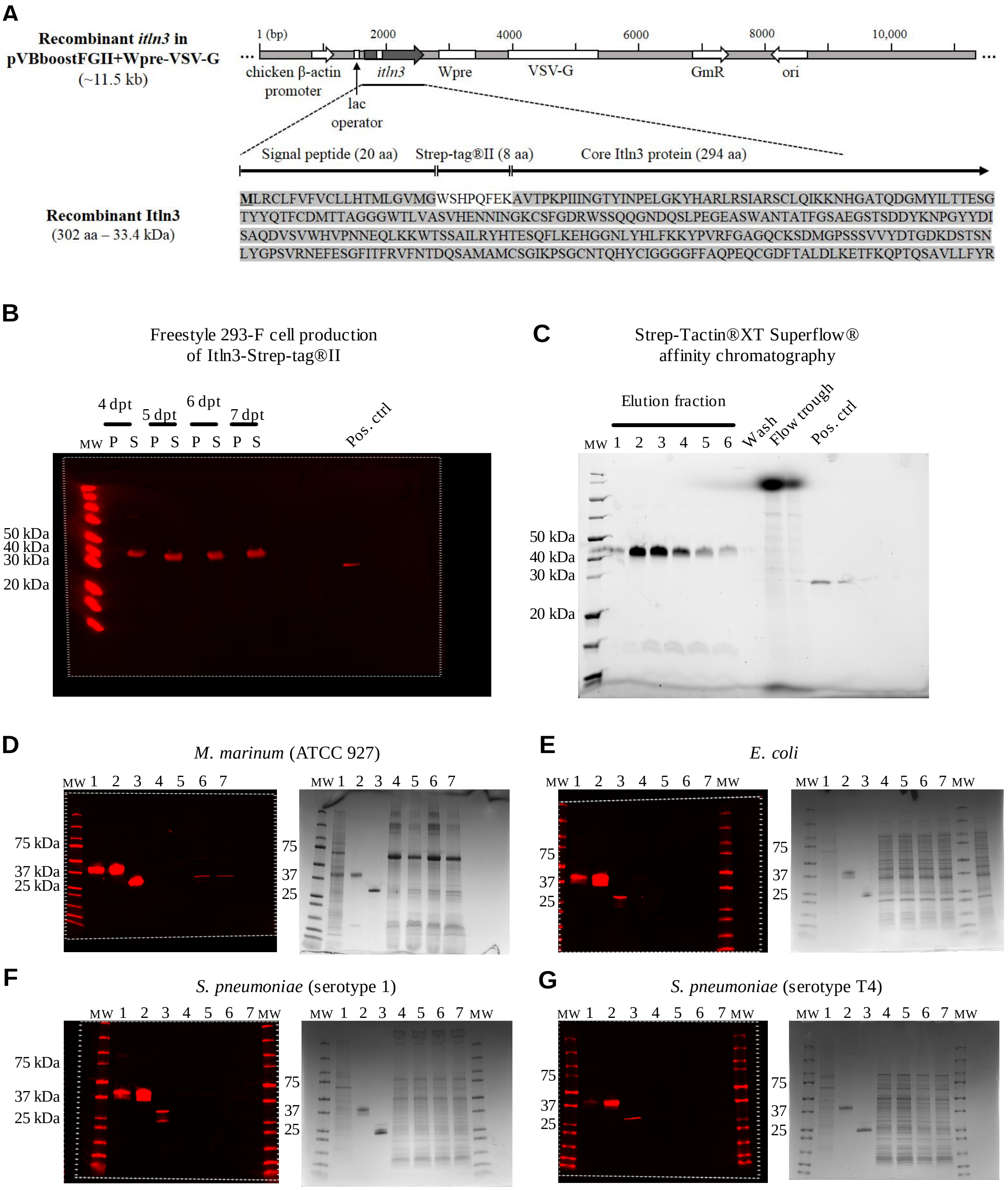

Supplementary Figure 7. Recombinant Itln3 does not bind to *M. marinum*, *S. pneumoniae* or *E. coli*. A) A schematic representation of the recombinant *itln3* cloned into pVBoostFGII+Wpre-VSV-G expression vector and of the expected 302 amino acid long Itln3-Strep-Tag®II protein product.

GmR = gentamycin resistance. ori = origin of replication. **B)** The recombinant Itln3 protein was produced in Freestyle 293-F cells, and an approximately 40 kDa product was detected with Western Blot between 4 and 7 days post transfection (dpt) in the supernatant (S) but not in the pellet (P). **C)** Following affinity chromatography with Strep-Tactin®XT Superflow® matrix, elution fractions 1-6 were run with SDS-PAGE. **D-G)** Recombinant Itln3-Strep-tag®II and GFP-Twin-Strep-tag® were incubated with either *M. marinum*, *E. coli* or *S. pneumoniae* (serotypes 1 and T4) in the presence (+$CaCl_2$) or absence of calcium (+EDTA). Samples were analyzed with SDS-PAGE and Strep-tagged proteins detected from blotted membrane at 700 nm using Strep-Tactin Oyster 645 conjugate. The lanes are numbered as follows; control samples: 1. 20 µl of Itln3 containing cell culture medium, 2. 1 µg of Itln3-Strep-Tag®II, 3. 1 µg of GFP-Twin-Strep-tag®, and the bacteria containing samples: 4. 7.5 µg of Itln3-Strep-Tag®II in binding buffer (+$CaCl_2$), 5. 7.5µg of Itln3-Strep-Tag®II in the absence of calcium (+EDTA), 6. 7.5µg of GFP-Twin-Strep-tag® in binding buffer (+$CaCl_2$), 7. 7.5µg of GFP-Twin-Strep-tag® in the absence of calcium (+EDTA). The PageRuler™ Unstained Broad Range Protein Ladder (#26630, Thermo Fisher Scientific) was used in panels B-C as a molecular weight marker (MW), whereas Precision Plus Protein™ Dual Xtra (#161-0377, Bio-Rad Laboratories) was used in panels D-G. White lines indicate the borders of the immunoblots. SDS-PAGE images are cropped to exclude portions that do not contain experimental samples. In panels D-G the same acquisition values for 700 nm channel have been applied.

Supplementary Table 1. Zebrafish (V3) Gene Expression Microarray, 4x44K (Agilent Technologies).

Up-regulated transcripts

Probe Name	Systematic Name	Gene Symbol	Gene Name	Average log2 Fold Change
A_15_P110361	NM_001004534	si:busm1-194e12.11 (mhc2)	si:busm1-194e12.11 (mhc2)	5.43
A_15_P269921	EH455384			4.40
A_15_P695731	ENSDART00000151988	LOC559001	fatty acid synthase-like	4.33
A_15_P541012				
A_15_P670021				
A_15_P286336	NM_001017909	zgc:112992	zgc:112992	4.24
A_15_P151116				
A_15_P772381	ENSDART00000054817	nansb	N-acetylneuraminic acid synthase b	4.11
A_15_P676036	NM_001139464	ela2	elastase 2	4.06
A_15_P437360	NM_214751	pck1	phosphoenolpyruvate carboxykinase 1 (soluble)	4.00
A_15_P661356	NM_198815	scd	stearoyl-CoA desaturase (delta-9-desaturase)	4.00
A_15_P727551				
A_15_P176621				
A_15_P170986	NM_001037420	ugt5a1	UDP glucuronosyltransferase 5 family, polypeptide A1	3.95
A_15_P117864	NM_001024408	ela3l	elastase 3 like	3.95
A_15_P623151	NM_131516	pvalb2	parvalbumin 2	3.90
A_15_P163756				
A_15_P208801	NM_001044323	zgc:152753	zgc:152753	3.88
A_15_P198561	NM_201173	cyp7a1a	cytochrome P450, family 7, subfamily A, polypeptide 1a	3.88
A_15_P100059	NM_001020482	cpa1	carboxypeptidase A1 (pancreatic)	3.84
A_15_P749196	NM_205572	pvalb1	parvalbumin 1	3.84
A_15_P106696				
A_15_P624116	NM_199605	zgc:66382	zgc:66382	3.79
A_15_P655936	NM_212783	pvalb4	parvalbumin 4	3.79
A_15_P132286				
A_15_P666139				
A_15_P209091	NM_001003737	zgc:92041	zgc:92041	3.72
A_15_P148011	NM_001076602	slc25a38a	solute carrier family 25, member 38a	3.69
A_15_P118321	NM_200212	pvalb3	parvalbumin 3	3.69
A_15_P664371				
A_15_P670531				
A_15_P104996	NM_001003423	tnni2b.2	troponin I, skeletal, fast 2b, tandem duplicate 2	3.68
A_15_P111799	NM_183070	sst1.1	somatostatin 1, tandem duplicate 1	3.66
A_15_P420075	TC425078			3.63
A_15_P162301	NM_001025180	c6ast4	six-cysteine containing astacin protease 4	3.62
A_15_P382820	NM_131105	tpma	alpha-tropomyosin	3.62
A_15_P754641				
A_15_P142756	NM_001020578	alox5b.3	arachidonate 5-lipoxygenase	3.59
A_15_P151326	NM_001003488	pkmb	pyruvate kinase, muscle, b	3.57
A_15_P674431	TC391723			3.57
A_15_P674096	NM_212618	ctrb1	chymotrypsinogen B1	3.51
A_15_P704401	ENSDART00000152233	si:ch1073-110a20.3	si:ch1073-110a20.3	3.51
A_15_P770551				
A_15_P681766	TC398257			3.49
A_15_P108203	NM_213131	mdh2	malate dehydrogenase 2, NAD (mitochondrial)	3.48
A_15_P620081	NM_200370	zgc:64043	zgc:64043	3.43
A_15_P170876	NM_001001893	zgc:113912 (mhc2)	zgc:113912 (mhc2)	3.40
A_15_P756000	NM_001004582	ctrl	chymotrypsin-like	3.35
A_15_P102930	NM_001045194	zgc:153896	zgc:153896	3.34
A_15_P728736	NM_001159584	itln3	intelectin 3	3.32
A_15_P190506				
A_15_P743431	NM_181653	tnnt3b	troponin T3b, skeletal, fast	3.31
A_15_P101676	NM_001003426	zgc:92745	zgc:92745	3.31
A_15_P488730	ENSDART00000122221	nfe2l2b	nuclear factor (erythroid-derived 2)-like 2b	3.28
A_15_P771326				
A_15_P624916	NM_001115089	myhz1.1	myosin, heavy polypeptide 1.1, skeletal muscle	3.25
A_15_P331879				
A_15_P657891				
A_15_P542682				
A_15_P665056	BC116547	myhz2	myosin, heavy polypeptide 2, fast muscle specific	3.24
A_15_P520672	NM_001003620	casq1a	calsequestrin 1a	3.24
A_15_P107326	NM_200516	zgc:66286	zgc:66286	3.23
A_15_P740921				
A_15_P764081	ENSDART00000126737	cd59	cd59 molecule	3.22
A_15_P545927				
A_15_P152786	NM_205575	tnni2a.3	troponin I, skeletal, fast 2a, tandem duplicate 3	3.22
A_15_P101653	NM_001002119	tpm2	tropomyosin 2 (beta)	3.22
A_15_P664046	AF539738	tnni2a.3	troponin I, skeletal, fast 2a, tandem duplicate 3	3.20
A_15_P729090	NM_001177498	ugt5a4	UDP glucuronosyltransferase 5 family, polypeptide A4	3.19
A_15_P657486	BC124207	wu:fc45h08	wu:fc45h08	3.19
A_15_P161846	NM_001007206	si:busm1-194e12.12 (mhc2)	si:busm1-194e12.12 (mch2)	3.19

A_15_P600272	ENSDART00000129396	LOC100536763	proline dehydrogenase 1, mitochondrial-like	3.18
A_15_P331241	NM_131619	mylz3	myosin, light polypeptide 3, skeletal muscle	3.18
A_15_P224231	NM_131563	tnnc2	troponin C type 2 (fast)	3.17
A_15_P150646	NM_001030262	plin2	perilipin 2	3.15
A_15_P107567				
A_15_P176531	NM_201009	aacs	acetoacetyl-CoA synthetase	3.15
A_15_P711187	ENSDART00000135295			3.14
A_15_P724331	NM_001110416	tnnt3b	troponin T3b, skeletal, fast	3.14
A_15_P762231	ENSDART00000151970			3.13
A_15_P133581	BC051151	muc5b	mucin 5b	3.13
A_15_P331464	NM_131188	mylpfa	myosin light chain, phosphorylatable, fast skeletal muscle	3.13
A_15_P510237				
A_15_P149696	NM_205678	myoz1a	myozenin 1a	3.10
A_15_P658631	NM_131727	sst2	somatostatin 2	3.08
A_15_P120396	NM_152982	myhz2	myosin, heavy polypeptide 2, fast muscle specific	3.07
A_15_P319166	NM_001271308	acaca	acetyl-Coenzyme A carboxylase alpha	3.07
A_15_P350165				
A_15_P106920	ENSDART00000146702	tnni2b.2	troponin I, skeletal, fast 2b, tandem duplicate 2	3.05
A_15_P661781	NM_001083827	col10a1	collagen, type X, alpha 1	3.03
A_15_P205041	NM_001076723	ugt5a2	UDP glucuronosyltransferase 5 family, polypeptide A2	3.03
A_15_P116208	NM_199271	cpa5	carboxypeptidase A5	3.02
A_15_P102410	NM_001003729	zgc:92137	zgc:92137	3.01

Down-regulated transcripts

Probe Name	Systematic Name	Gene Symbol	Gene Name	Average log2 Fold Change
A_15_P558307	ENSDART00000110325	si:ch211-160b11.4	si:ch211-160b11.4	-3.01
A_15_P163501	NM_001098254	si:dkey-11f4.16 (cd58)	si:dkey-11f4.16 (cd58 molecule)	-3.20
A_15_P114150	NM_199856	tdo2b	tryptophan 2,3-dioxygenase b	-3.21
A_15_P117407	ENSDART00000173485.2	ENSDARG00000093796 (itln2-like)	intelectin 2 -like	-3.22
A_15_P299736	EH440031			-3.35
A_15_P132151	NM_212779	mpx	myeloid-specific peroxidase	-3.42
A_15_P158486				
A_15_P637411				
A_15_P164866	ENSDART00000018062	cfbl	complement factor B-like	-3.42
A_15_P144444	NM_001159541	itln2	intelectin 2	-3.47
A_15_P628696	NM_001001730	cyp51	cytochrome P450, family 51	-3.74
A_15_P727566	NM_001089487	zgc:162608	zgc:162608	-3.76
A_15_P176481				
A_15_P181626	BC134957			-3.85
A_15_P678601	EB975183			-3.98
A_15_P365355	NM_001030096	tgm1l4	transglutaminase 1 like 4	-4.02
A_15_P631151				
A_15_P731581	TC444503			-4.10
A_15_P529217				
A_15_P163579	NM_001007167	si:busm1-266f07.1 (mhc2)	si:busm1-266f07.1 (mhc2)	-4.28
A_15_P176991	NM_001200021	LOC570474	5-hydroxyisourate hydrolase-like	-4.59
A_15_P118284	NM_001077607	irg1l	immunoresponsive gene 1, like	-4.72
A_15_P695546	EH492309	si:ch211-66i15.5	si:ch211-66i15.5	-4.73
A_15_P205021	NM_001040359	zgc:136902	zgc:136902	-4.78
A_15_P357500	TC431869			-4.78
A_15_P309606	ENSDART00000064720	tgm1	transglutaminase 1	-4.94

Supplementary Table 2. Enriched processes from gene ontology analysis.

Up-regulated transcripts vs. background list.

GO term	Description	P-value
GO:0006936	muscle contraction	3.90E-6
GO:0003012	muscle system process	5.47E-6
GO:0006941	striated muscle contraction	8.58E-6
GO:0060048	cardiac muscle contraction	4.57E-5
GO:0003009	skeletal muscle contraction	6.05E-5
GO:0006508	proteolysis	7.21E-5
GO:0061959	response to (R)-carnitine	1.65E-4
GO:0072330	monocarboxylic acid biosynthetic process	2.35E-4
GO:0019882	antigen processing and presentation	2.78E-4
GO:0060359	response to ammonium ion	3.06E-4
GO:0032787	monocarboxylic acid metabolic process	3.38E-4
GO:0044283	small molecule biosynthetic process	3.60E-4
GO:0034284	response to monosaccharide	4.90E-4
GO:0033273	response to vitamin	4.90E-4
GO:0009743	response to carbohydrate	4.90E-4
GO:0009746	response to hexose	4.90E-4
GO:0009749	response to glucose	4.90E-4
GO:0097435	supramolecular fiber organization	6.12E-4
GO:0030239	myofibril assembly	7.05E-4
GO:0055092	sterol homeostasis	7.15E-4
GO:0042632	cholesterol homeostasis	7.15E-4
GO:0014823	response to activity	9.82E-4

Down-regulated transcripts vs. background list.

GO term	Description	P-value
GO:0051707	response to other organism	1.95E-6
GO:0051704	multi-organism process	2.70E-6
GO:0043207	response to external biotic stimulus	4.13E-6
GO:0009607	response to biotic stimulus	4.83E-6
GO:0009617	response to bacterium	2.58E-5
GO: 0006695	cholesterol biosynthetic process	3.51E-5
GO:1902653	secondary alcohol biosynthetic process	3.51E-5
GO:0009605	response to external stimulus	3.56E-5
GO:0044281	small molecule metabolic process	6.15E-5
GO:0016126	sterol biosynthetic process	1.26E-4
GO:1902652	secondary alcohol metabolic process	1.76E-4
GO:0008203	cholesterol metabolic process	1.76E-4
GO:0046165	alcohol biosynthetic process	2.21E-4
GO:0016125	sterol metabolic process	3.74E-4
GO:0006694	steroid biosynthetic process	4.39E-4
GO:0006952	defense response	4.58E-4
GO:0010310	regulation of hydrogen peroxide metabolic process	6.11E-4
GO:0043152	induction of bacterial agglutination	6.11E-4
GO:0033488	cholesterol biosynthetic process via 24,25-dihydroanosterol	6.11E-4
GO:1901617	organic hydroxy compound biosynthetic process	7.06E-4
GO:0042742	defense response to bacterium	7.94E-4
GO:0008202	steroid metabolic process	8.87E-4

Supplementary Table 3. Primers used in the qPCR analyses.

Gene	Ensembl Identifier	Sequence 5´-3´	Reference
itln1	ENSDARG00000007534	F GACGACTACAAGAACCCTGG<br>R ATCGTTGCATGTACCTATGCC	-
itln2	ENSDARG00000036084	F TATGGGAATGGCTGCCTTTC<br>R TTTCAAGCTCATGGTTGCTG	-
itln2-like	ENSDARG00000093796	F ACTGTTCAAGAAATTCCCTGTG<br>R ATGCCCAGTTGGTTTAGTGC	-
itln3	ENSDARG00000003523	F GTGCAACACAGGATGGCATG<br>R TTCTGCACTGCCAAACGTAG	-
eef1a1l1 (ef1a)	ENSDARG00000020850	F CTGGAGGCCAGCTCAAACAT<br>R ATCAAGAAGAGTAGTACCGCTAGCATTAC	(94)
mmits	-	F CACCACGAGAAACACTCCAA<br>R ACATCCCGAAACCAACAGAG	(56)

Supplementary methods

Experimental *S. pneumoniae* infections

S. pneumoniae serotype 4 (T4, sequence type 205) as well as serotype 1 (sequence type 306) culture and infections were performed as described previously[1, 2]. In brief, *S. pneumoniae* were streaked on 5 % lamb blood agar plates (Tammer-Tutkan Maljat, Tampere, Finland) and cultured overnight. In the morning the bacteria were inoculated into 5ml Todd Hewitt broth (Becton, Dickinson and Company, New Jersey, USA) supplemented with 0.5% Todd-Hewitt yeast extract (Becton, Dickinson and Company) and cultured until OD620 ≈ 0.40. 2-day-old zebrafish embryos were anesthetized with 0.02% 3-amino benzoic acid ethyl ester (Sigma-Aldrich) and microinjected into the blood circulation valley using 2 nl of bacteria suspended in 0.2 M potassium chloride (KCl) with 7 mg/ml of tetramethylrhodamine dextran (Thermo Fisher Scientific) to visualize the injections. The survival of the embryos were monitored at least twice a day before 50 hours post infection (hpi) and at least once a day between 2 and 5 dpi. *S. pneumoniae* counts (CFU) in each of the infections were counted by plating inoculates of bacteria on 5% lamb blood agar plates overnight at 37°C with 5% CO_2.

Recombinant Itln3 production

Recombinant zebrafish *itln3* gene was initially synthetized by GeneArt (Invitrogen). AttB-sites for Gateway cloning were added with PCR using the following primers F: GGGGACAAGTTTGTACAAAAAAGCAGGCTTCACCATGCTGCGCTGCCTGTTTGTCTTCG and R: GGGGACCACTTTGTACAAGAAAGCTGGGTTTTATTAACGATAAAACAGCAGAACTG CGCTC. PCR product was Gateway cloned into a donor plasmid pDONR™211 (Thermo Fisher Scientific) and subsequently into the mammalian expression plasmid pVBboostFGII-Wpre-VSV-G[3], and gene of interest was verified by sequencing. Freestyle 293-F cells (Thermo Fisher Scientific) were

transiently transfected with 10 µg of expression plasmid using FreeStyle™ MAX Reagent (Thermo Fisher Scientific). Cells were cultured for a total of 7 days and both the cells and medium were collected for analysis and protein purification. Itln3 containing an N-terminal Strep-tag®II (Itln3-Strep-tag®II) was purified with Strep-Tactin® XT resin (IBA Life Sciences, Göttingen, Germany) using excess of biotin for elution. Purity and the molecular size of the Itln3-Strep-tag®II was analyzed by SDS-PAGE and Western blot, respectively. Briefly, the protein containing fractions were ran with SDS-PAGE (4-20% gradient gel, Bio-Rad Laboratories) or blotted from the gels on nitrocellulose membrane using Trans-Blot® Turbo™ transfer system (Bio-Rad Laboratories). SDS-PAGE gels were stained with PageBlue™ protein stain (Thermo Fisher Scientific). Immunoblot membranes were blocked in 1% BSA in 0.05% Tween TBS for 30 min at room-temperature (RT) and washed three times with 0.05% Tween TBS for 5 min. Strep-tagged proteins on the blots were detected with 2.5µg of Streptactin Oyster 645 conjugate (IBA Life Sciences, Göttingen, Germany) in 5ml of 0.05% Tween TBS and incubated for 1 h at RT. SDS-PAGE images were obtained with ChemiDoc™ XRS+ system (Bio-Rad Laboratories) and analyzed with Image Lab software (v. 5.2; Bio-Rad Laboratories), whereas blot imaging was done with the Odyssey® CLx (LI-COR Biosciences, Nebraska, USA) at 700 nm at 169 µm resolution, and the images were analyzed by using the Image Studio Lite (v. 5.2; LI-COR Biosciences).

Bacterial binding assay

M. marinum (ATCC 927) and *S. pneumoniae* (T4 and S1) were cultured as described above. *S. pneumoniae* was cultured until OD620 = 0.25 (serotype S1) or 0.35 (serotypeT4), whereas *M. marinum* was cultured until OD600 = 0.614 and 1 ml of the culture used for the bacterial binding assay. *E. coli* was inoculated from a glycerol stock into 5 ml of LB, 225 rpm 37°C. 100 µl of the culture (OD600 =1.614) was pelleted 3 min 10 000 g. Bacterial pellets were washed with 1 ml of sterile PBS, pelleted again 3 min 10 000 g and 1 ml of the protein samples added on the pellet for the bacterial binding. For

this 7.5 µg of the Itln3-Strep-tag®II or 7.5 µg of the GFP-Twin-Strep-tag® negative control protein (IBA Life Sciences) were used in either 1 ml of the binding buffer (20 mM HEPES, 150 mM NaCl, 10 mM $CaCl_2$, 0.1% BSA, 0.05% Tween-20, pH 7.4) or in the EDTA containing buffer (20 mM HEPES, 150 mM NaCl, 10 mM EDTA, 0.1% BSA, 0.05% Tween-20, pH 7.4). The samples were incubated 2 h in rotation at 4°C and washed with 1 ml of PBS as above. The samples were suspended in 30 µl of PBS, and 10 µl of 4xSDS sample buffer (0.2 M Tris-HCl pH 6.8, 8% SDS (w/v), 40% glycerol, 4% β-mercaptoethanol, 0.05 M EDTA, 0.8 mg/ml bromophenol blue) and incubated 10 min 98°C with frequent vortexing. Before loading onto the gel, samples were spinned to pellet bacterial debris and approximately 15 µl of the supernatant was run on two mini-Protean TGX-gels (Bio-Rad Laboratories, California, USA). One gel was stained over night with Page Blue™ Protein Staining Solution (Thermo Fisher Scientific) and the other gel was blotted using the Trans-Blot Turbo Transfer System (Bio-Rad Laboratories). The blot was prepared for imaging and imaged as mentioned above. 1 µg of the Itln3-Strep-tag®II, 1 µg the GFP-Twin-Strep-tag® (IBA Life Sciences) and 20 µl of the Itln3 containing medium from the cell culture were used as positive controls on the gel.

Supplementary references

1. Rounioja, S. *et al.* Defense of zebrafish embryos against Streptococcus pneumoniae infection is dependent on the phagocytic activity of leukocytes. *Dev. Comp. Immunol.* **36**, 342-348 (2012).

2. Saralahti, A. *et al.* Adult zebrafish model for pneumococcal pathogenesis. *Dev. Comp. Immunol.* **42**, 345-353 (2014).

3. Heikura, T. *et al.* Baculovirus-mediated vascular endothelial growth factor-D(ΔNΔC) gene transfer induces angiogenesis in rabbit skeletal muscle. *J Gene Med* **14**, 35-43 (2012).
